# Evidence for increased background neural noise in migraine with aura: Hyperactive but not hyperresponsive

**DOI:** 10.1111/head.15046

**Published:** 2025-09-16

**Authors:** Louise O'Hare, Paul B. Hibbard, Arnold J. Wilkins

**Affiliations:** ^1^ Nottingham Trent University Nottingham UK; ^2^ University of Stirling Stirling UK; ^3^ University of Essex Colchester UK

**Keywords:** contrast, electroencephalography, noise, precision ophthalmic tints, sweep visual evoked potential

## Abstract

**Objective:**

This study had two aims: (1) to investigate the contrast gain in migraine and compare it to that in photosensitive epilepsy; (2) to explore any effects of colored spectacles (precision ophthalmic tints [POT]) on contrast gain.

**Background:**

Individuals with migraine with aura (MA) typically show high amplitude electrophysiological responses, but poor performance on visual tasks. One possible explanation is increased neural “noise” in the visual cortex in MA. “Noise” is neural activity that does not carry information about the stimulus.

**Methods:**

This is a case–control study of individuals with MA and controls with no family history of migraine, as there is a tendency for migraine to run in families. We measured the steady‐state visual evoked potential in response to a sinewave grating (striped pattern) that varied in contrast (appeared to flicker on and off) at 5 and 17 Hz in 15 MA and 15 control participants. The maximum contrast (stimulus intensity) increased progressively from 10% to 90% in nine equal steps. We also measured the effect of colored spectacles (POTs) on the electroencephalogram (EEG) response. The experiment was a mixed factorial design, with one between‐participants factor (experimental vs. control group) and two within‐participants factors (contrast and lens type [none, POT, or control]). The dependent variables were the steady‐state visual evoked potential response, and the background EEG activity. In experiment 2, discomfort judgments on a rating scale of 0–9 from a separate set of 12 MA and 12 control participants were also collected during the EEG session. Data were collected between February 2022 and August 2024.

**Results:**

At the faster flicker rate of 17 Hz (appearing on and off 17 times per second), the electrophysiological response of the MA group showed increased background activity (EEG power at frequencies other than the stimulation frequency) (experiment 1: mean for the migraine group = −666.45 dB/Hz, standard error [SE] = 116.69; mean for the control group = −1100.50 dB/Hz, SE = 164.99; coefficient estimate = 434.09, *p* = 0.016, confidence interval [CI], 82.24–735.94; estimated Cohen's *d* of 0.94, CI, 0.14–1.73; experiment 2: migraine group mean = −500.01 dB/Hz, SE = 122.99; control group mean = −741.88 dB/Hz, SE = 126.12; coefficient estimate = 265.04, *p* = 0.049, CI, 1.52–528.56, estimated Cohen's *d* of 0.95, CI, 0.05–1.84). The increase in EEG power with contrast at the stimulation frequency was similar in both MA and control groups. The MA group experienced more discomfort compared to the control group (median rating for migraine group = 4, interquartile range [IQR] = 4, median rating for control group = 3, IQR = 3, coefficient estimate = 3.58, *p* = 0.003, CI, 1.18–6.00) and faster flicker (17 Hz) was judged more uncomfortable than slower flicker (5 Hz) by both groups (median rating for 5 Hz = 3, IQR = 3, median rating for 17 Hz = 4, IQR = 4, coefficient estimate = 0.97, *p* < 0.001, CI, 0.49–1.45). There was no specific reduction in EEG response in the MA group compared to controls with POTs.

**Conclusions:**

The increased EEG responses in MA show evidence that in migraine the brain is “noisier” compared to controls. As the contrast response was similar in both MA and control groups, this suggests typical contrast gain control, as distinct from the abnormality seen previously in photosensitive epilepsy. The chosen color of POTs can reduce discomfort judgments under some circumstances, although this does not appear to be specific to MA.

## Plain Language Summary

People with migraine with aura (MA) and people with photosensitive epilepsy have similarities in terms of their sensitivities to visual stimuli. In this study, we used electrophysiological (EEG) recordings of brain activity in people with MA to determine if low‐level visual processing is similar to that in people with photosensitive epilepsy. We found evidence for different mechanisms of visual sensitivity in MA compared to photosensitive epilepsy, and there was consistent evidence of increased background brain activity in MA compared to people without migraine, suggesting greater neural “noise” in people who have MA.

**Abbreviations**
ANOVA: analysis of variance
CI: confidence interval
CRT: cathode ray tube
EEG: electroencephalography
fMRI: functional magnetic resonance imaging
IHS: International Headache Society
MA: migraine with aura
POT: precision opthalmic tints
SE: standard error
SSVEP: steady‐state visual evoked potential

## INTRODUCTION

Migraine is a sensory processing disorder^1^ that affects vision. Pattern‐induced visual discomfort is higher in migraine interictally,^2^ and visual stimuli can trigger attacks.^3^, ^4^, ^5^ Migraine with aura (MA) is characterized by sensory hallucinations (and in some subtypes, motor and language disturbances). These sensory hallucinations occur before, or at the time of the onset of the headache. The sensory hallucinations are most commonly visual.^6^, ^7^

There is convergent evidence that individuals with migraine have a “hyperexcitable” visual cortex.^8^, ^9^ There are larger visually evoked potentials (VEP),^10^ larger functional magnetic resonance imaging (fMRI) responses,^11^ and greater ease with which perceptual and behavioral responses can be induced using transcranial magnetic stimulation.^8^ Individuals with migraine are unusually averse to flicker, consistent with an overly responsive visual brain.^12^ However, reduced visual performance, such as poorer contrast detection, orientation discrimination, and motion sensitivity, is found in migraine.^13^, ^14^, ^15^, ^16^, ^17^ Hyperexcitability in migraine is thus associated with impaired visual function.

**FIGURE 1:**
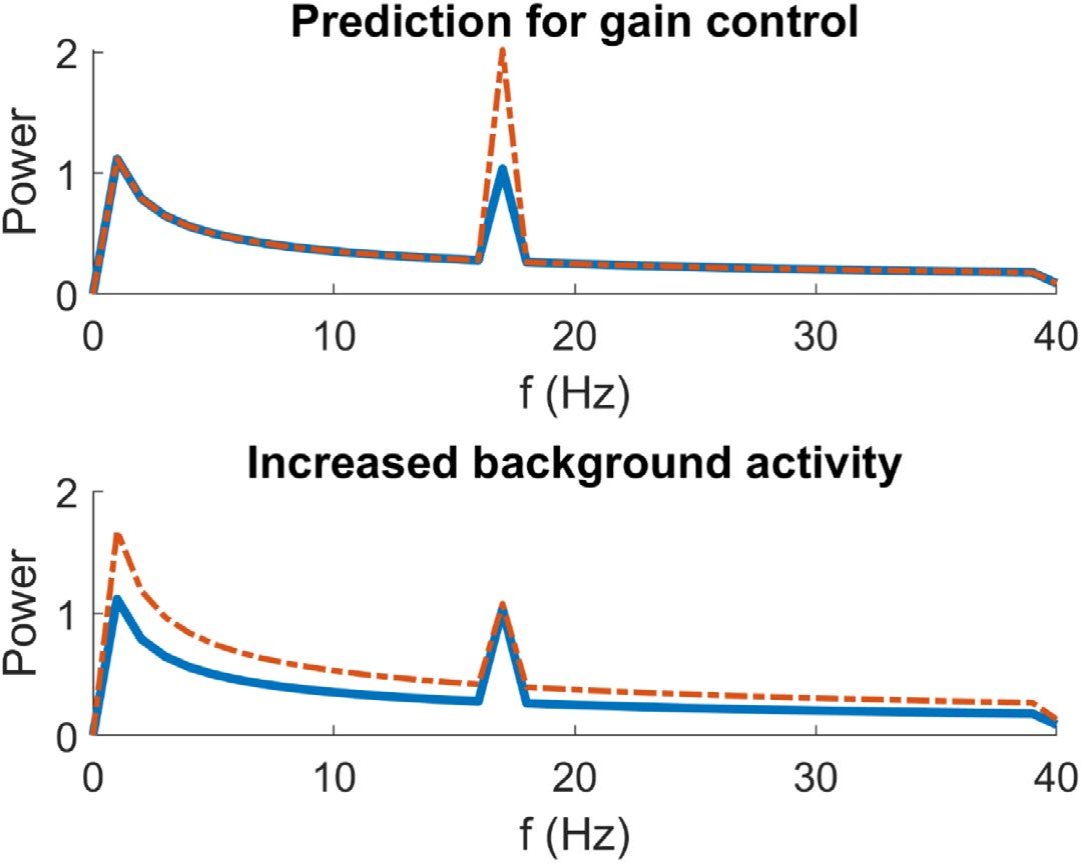
Schematic diagram of the predictions of the experiment. In the top figure, the prediction for the power spectrum for contrast gain control is demonstrated. The *x*‐axis shows the frequency (Hz), and the *y* axis shows the Power, as this is a schematic diagram then no particular units are associated. Compared to the blue solid line, the red broken line shows an increased SSVEP response to contrast at 17 Hz. This increased response will saturate at a high contrast level, and not increase further. If there is a lack of contrast gain control, the response to increasing contrast will continue to increase. The lower figure shows the demonstration of increased background activity. This shows a higher amplitude spectrum overall for all frequencies, as well as the stimulation frequency of 17 Hz. The magnitude of the response to stimulation is no higher for the blue compared to the red line, if the baseline response is accounted for. Abbreviation: SSVEP, steady‐state visual evoked potential. [Color figure can be viewed at wileyonlinelibrary.com]

Neural activity in brain areas responsive to visual input consists of both “signal” and “noise” components.^16^ The signal is the direct response to the current visual input—and will tend to increase in amplitude as the strength of this input, such as its contrast, is increased. The contrast gain function describes the way in which the response increases with an increase in stimulus contrast. The noise component is the randomly fluctuating background activity (Figure 1). Hyperexcitation in migraine could take the form of an increase in signal, noise, or both. It is therefore useful to distinguish between “hyperactivity” (referring to the overall increase in level of neural activity, whether signal or noise) and “hyperresponsiveness” (an increase in signal only).

Cortical hyperactivity in migraine could reflect an in increase in contrast gain, an increase in neural noise, or a combination of the two. The generally poorer task performance in people with migraine is consistent with an increase in noise.^17^, ^18^, ^19^ However, in individuals with photosensitive epilepsy, Porciatti et al.,^20^ showed stronger signal responses, attributable to a reduction in contrast gain control. In healthy controls, the amplitude of the steady‐state visual evoked potential (SSVEP) increases with contrast up to 20% but saturates at this level. In patients with photosensitive epilepsy, responses continued to increase with contrast above this value, leading to much greater responses to high contrast stimuli. Contrast gain control can help prevent hyperactivity in response to a visual stimulus. In this study, we investigated whether hyperexcitation in migraine could be characterized as a similar reduction in the gain control, and thus greater responses to high‐contrast stimuli.

We also investigated whether precision ophthalmic tints (POTs) affected the visual response. POTs are specific, individually chosen colored filters that can reduce visual discomfort. They are dispensed either as overlays to help with discomfort from text, or as glasses to be worn more generally. People with headache have reported reduced headache frequency with POTs.^21^ Huang et al.^11^ showed that POTs reduced fMRI blood oxygenation level dependent responses to aversive patterns in individuals with migraine. POTs are thought to reduce the level of cortical excitability, which is greater in migraine.^22^ Here, we test whether any reduction in gain control that may be found in people with migraine can be reduced with POTs. Our hypotheses for the study were pre‐registered at the Open Science Framework (https://osf.io/g2kqd). The specific hypotheses were (1) people with MA will show a different contrast response function compared to controls, with a failure to saturate at high contrasts, indicating reduced contrast gain control; and (2) this reduction in contrast gain control in migraine will be normalized with POTs.

## METHODS

### Experiment 1

#### Observers

This was a case–control study. There were two parts to the study. Experiment 2 was to replicate experiment 1 with a separate set of participants and slightly stronger stimuli, as they were well‐tolerated, but too weak in experiment 1. All experiments adhered to the Declaration of Helsinki and were approved by Nottingham Trent University School of Social Science Ethics Committee (ethics application no. 2021/408). Participants were recruited from staff and students at Nottingham Trent University by word of mouth, poster, and internal messages. All participants gave written informed consent to take part in the study. All participants reported normal or corrected‐to‐normal vision. The MA group was recruited based on fulfilling the International Headache Society Classification Criteria^23^ for MA. The criteria consisted of at least two attacks involving fully reversible aura symptoms, and at least three of the six listed aura characteristics (gradual spreading, occurring in succession, lasting 5–60 min, unilateral location, one positive symptom, accompanied or followed by headache in the next hour). The exact manifestation of the aura varies between individuals, but common patterns include flashes of light (phosphenes), shimmering zig‐zag patterns (“scintillating scotoma”), and other hallucinations.^24^ Control participants were individuals with no family history of migraine and no headaches. This included any of the primary headache disorders (e.g., tension‐type headache, cluster headache, migraine without aura, etc.), as well as any other regularly occurring headaches, explained or otherwise. It was acceptable that participants in the control group may have experienced a small number of headaches in the past year due to illness, etc., but this was to be the exception. This was based on self‐report. Participants were not excluded on the basis of medication, but any participant experiencing a migraine attack, or had experienced an attack within 2 days, was asked to reschedule. People with photosensitive epilepsy were excluded.

#### Apparatus

Stimuli were displayed on a 19‐inch Mitsubishi Diamond Pro 920 CRT display with Windows 10 operating system running MATLAB version 2020b (The MathWorks Inc., Natick, MA, USA) and the Psychtoolbox plugins.^25^, ^26^, ^27^ Screen resolution was 1024 × 768 pixels with an 85 Hz refresh rate. The display was calibrated using a Minolta L110 photometer. EEG recordings were made using the Biosemi 64‐channel system and Actiview acquisition software. Data were referenced to the linked mastoids, and eye movement channels were recorded using electrodes on the infraorbital and outer canthi. POTs were chosen using an Intuitive Colorimeter Mark 2 (Cerium Visual Technologies, Tenterden, Kent). The Intuitive Colorimeter is a device that enables a participant to systematically vary hue and then saturation of colors, while viewing a stimulus (in this case text) to see which color, if any, makes the stimulus more comfortable to look at. The chosen color is replicated in the POT. The POTs were spectacle lenses worn over any existing correction. The placebo lens was estimated by determining a chromaticity with the same saturation (suv) as the POT but differing in chromaticity by approximately 0.06. The units for the saturation value (suv) refer to the CIE LUV 1976 colour system and represent the distance from the white point. This results in two points, with chromaticity either side of the chromaticity of the chosen POT. One of these was selected at random for the control lens. There was some variation in transmission of the lenses due to a mismatch between the light sources of the screen and the Colorimeter, which could be considered as “bluer” and so affected the estimate of the appropriate control lens. This resulted in the POT having on average a greater transmission compared to the control lenses. Therefore, transmission is included as a covariate in the linear mixed effects models used for analysis. The chromaticities and transmissions of POT and control lenses can be seen in the Supporting Information Part 4.

#### Stimuli

**FIGURE 2:**
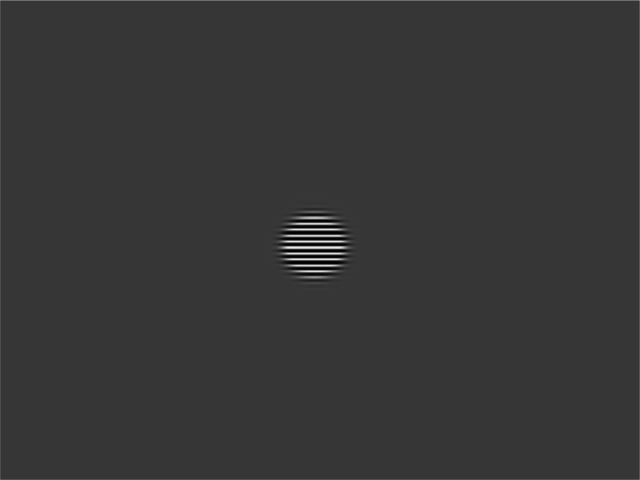
Schematic diagram of the stimulus.

Stimuli were presented on a mid‐gray background with a black fixation cross. A schematic diagram of the stimulus can be seen in Figure 2. Sine‐wave gratings (striped patterns) were presented in a 3° diameter (visible section) Gaussian window with a roll‐off (sigma) of 10 pixels. The gratings had a spatial frequency of two cycles/degree and were ramped in contrast between the maximum contrast level and 0% (indistinguishable from the background) with a sine‐wave temporal profile. This appeared as a smooth increase in intensity before fading away to gray again. Because this appearance and disappearance of the stimulus was rapid, this appeared as flicker, for 2 s at either 5 or 17 Hz before the contrast increased to the next level. There were nine contrast levels between 10% and 90% Michelson contrast. Stimuli were displayed for 18 s for each trial (2 s for each level of contrast). There were 10 repetitions of the 18‐s trial for each flicker frequency, with the exception of the first three participants (all MA) who had only five repetitions. This was due to technical issues with the recordings and may have resulted in less reliable estimates for these three participants. Flicker frequencies were randomly interleaved. No participant reported any adverse effects during or after the experiment, despite being encouraged to do so if these occurred.

#### Procedure

Observers were seated in a quiet darkened room approximately 35 cm from the display. No chinrest was used. Participants viewed the stimuli binocularly and were instructed to look at the center of the pattern and keep as still as possible. EEG was sampled at 2048 Hz and down‐sampled offline to 256 Hz.

#### Analysis

Analyses were conducted using MATLAB version 2020b (MathWorks Inc.) with the Statistics and Machine Learning Toolbox, and R version 4.0.5 (2021‐03‐31) (R Foundation for Statistical Computing, Vienna, Austria) with the “ordinal” package (version 2019.12‐10). This is the primary analysis of these data: they have not been previously published. Descriptive statistics were presented as means and standard errors (SEs) for normally distributed continuous variables, medians, and interquartile ranges (IQRs) for ordinal variables.

The planned analysis was pre‐registered at the Open Science Framework (https://osf.io/3w89b/). We planned to analyze (1) the EEG response to contrast in MA and control groups; and (2) the effect of POTs on the EEG response. There was some deviation from the original preregistration, specifically the analysis method. It was felt that linear mixed effects models were a more justifiable analysis method compared to the ANOVA in the original article by Porciatti et al.^20^ This is primarily because ANOVA does not accommodate the continuous nature of contrast as a predictor. In addition, a mixed effect model better captures the variation between individuals and is better able to tolerate missing data due to EEG artifact rejection. In addition, *post hoc* analysis of the incidental finding of group differences in background activity was also included. This was not in the preregistered report.

EEG data were band‐pass filtered using a 0.1 to 40 Hz finite impulse response filter to remove drift and line noise artifacts. Data were divided into 18‐s epochs corresponding to a single trial, and the baseline was removed from the first 500 ms of each trial. Data from each trial were then divided into 2‐s epochs corresponding to the contrast level. Bad channels were removed using an automated threshold procedure—first estimating the joint probability of the channels and rejecting those channels 5 standard deviations from the mean. Missing channels were interpolated. Gratton‐Coles^28^ procedure was used to correct for eye movements, using a blink criterion of 200 mV threshold in a 20‐ms period.

**FIGURE 3:**
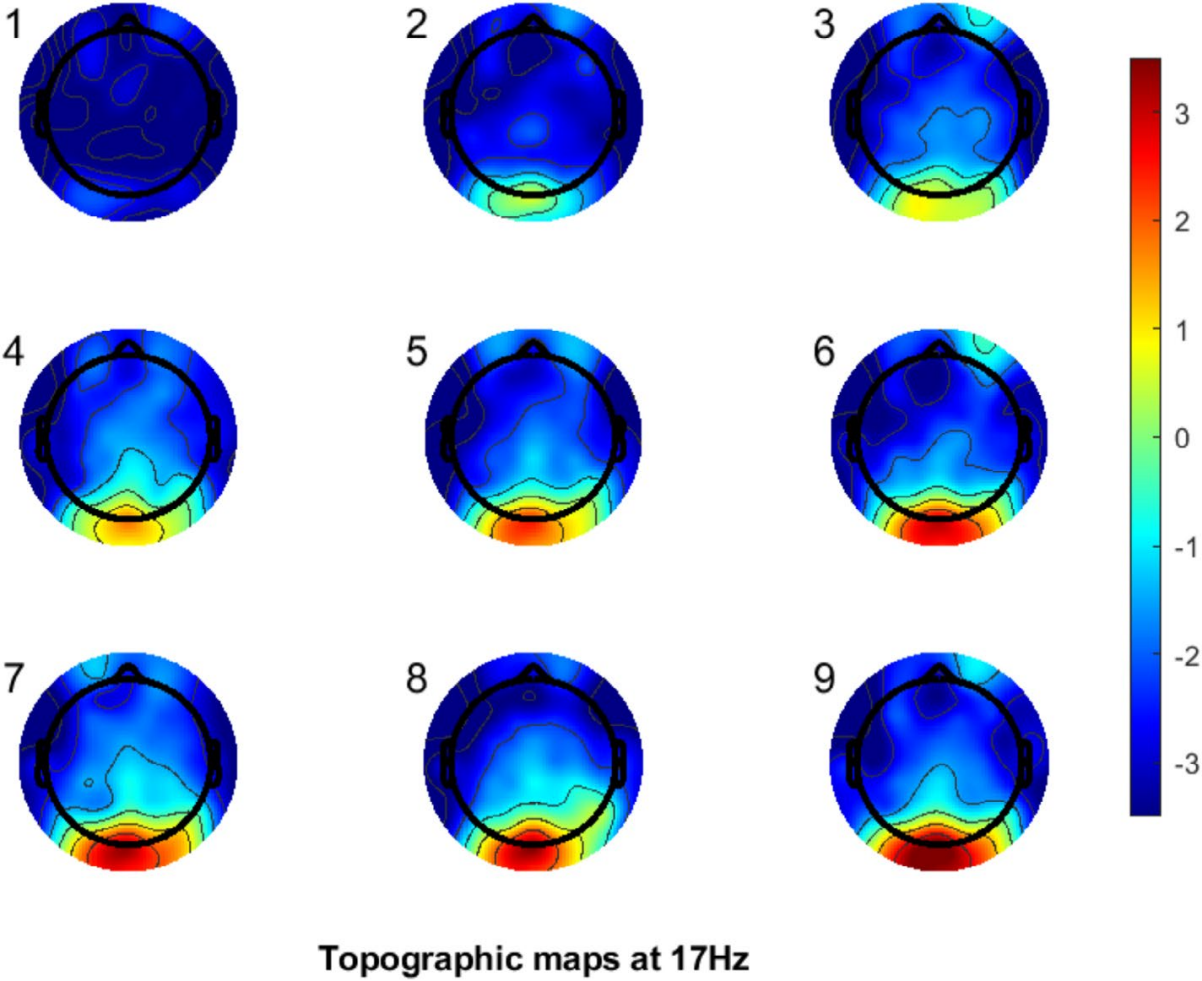
Scalp topography of electroencephalogram response to 17 Hz flicker in dB/Hz. Each individual scalp topography plot is for each individual level of contrast, 1 refers to 10% contrast, through to 9 at 90% contrast. [Color figure can be viewed at wileyonlinelibrary.com]

Welch's method of spectral analysis (using the MATLAB function “pwelch”) was performed on the cleaned data using the default Hamming filter, with a window length of 512 samples, 0% overlap, and 512 discrete Fourier transform points. The resulting power spectral density was expressed in decibels (10 × log10). The power spectral density was estimated for each trial, and then averaged over trials. Figure 3 shows the distribution of power over the scalp for the 17 Hz stimulus. As expected, the occipital channels show greatest activity, and so channels O1, O2, Oz, and Iz were averaged as the channels of interest. There was insufficient signal from the 5 Hz stimuli to analyze, see Supporting Information Part 1.

Because there are differences in the recorded background level of activity between observers, a normalised SSVEP was used. This was the signal‐to‐noise ratio (SSVEP) of the response, estimated by dividing by the average response at 17 Hz by the response ~2 Hz above and below, so for 17 Hz the response was divided by the average of the response at 15–16 and 18–19 Hz.^29^ The SSVEP of the response was estimated for each level of contrast. In addition, the background activity level was estimated by calculating the total power between 6 and 30 Hz for all trials, omitting the 17 Hz stimulation frequency.

Outlying data points were identified and removed using the “isoutlier” function in MATLAB, which removes data points more than three absolute deviations from the median value. SSVEP of the responses were analyzed using a linear mixed effect model using the function “fitglme” in MATLAB. SSVEP was predicted using contrast, group, lens, and their interactions as fixed effects, and transmission of the lens as a covariate and observer as a random effect (intercept and slope model). Data were slightly skewed compared to a normal distribution (0.60), but these models are robust to slight skewness.^30^ Figures showing these distributions, homoscedasticity as well as the model selection process (used to address the trade‐off between the model fit and the number of parameters) can be seen in the Supporting Information Part 2. There was data loss of 13.33% for the MA observers and 10% data loss for the control participants across all lens conditions. In addition, outlying data points were excluded. In experiment 1, at 17 Hz, one outlier was identified. For experiment 2, there was one outlier from the 5 Hz stimulation, and eight outliers from the 17 Hz stimulation condition. Regression coefficients and their SEs are reported as unstandardized effect sizes as these are in the original units of measurement.^31^ To facilitate comparison with other literature, Cohen's *d* effects sizes are also reported for all main effects. Statistical testing was two‐tailed and considered significant at *p* < 0.05.

## RESULTS

Fifteen individuals with MA (six male, mean age = 29.8, SD = 12.7) and 15 controls (6 male, mean age = 26.8, SD = 8.2) participated in the study. Efforts were made to recruit participants similar in age and sex to the MA group; however, there was a slight variation in ages. MA participants fulfilled the criteria for MA, with one exception fulfilling the criteria for aura without headache. The overall pattern of results remained the same whether the individual with aura without headache was included in the analysis or not (see Supporting Information Part 3). Migraine characteristics can be seen in Table 1.

**TABLE 1:** Migraine group clinical characteristics for the participants in experiment 1.

Sex	Age	Glasses	Attack frequency (per month)	Moderate to severe pain	Other sensory aura	Language aura	Pro diagnosis	Duration (years)	Time since last attack (days)	Visual trigger
Female	23	Long‐sighted	1–3	Yes	Yes	Yes	MA	18	1 month	Yes
Female	29	Near‐sighted in right eye, far‐sighted in left eye	1–3	Yes	Yes	Yes	MA	13	2 weeks	Yes
Male	25	No	<1	Yes	No	No	No	11	Na	No
Female	22	Concentration	1–3	yes	Yes	No	MA	10	1 month	Yes
Male	25	No	<1	Yes	No	No	No	Na	1 year	No
Female	30	Short‐sighted	3–10	Yes	Yes	Yes	MA	7	5 days	Yes
Male	24	For migraine	<1	Yes	No	No	No	11	2 months	Yes
**Male**	**68**	**No**	**3–10**	**No**	**No**	**No**	**No**	**Na**	**4 days**	**Yes**
Female	20	No	1–3	Yes	No	Yes	MA	7	1 year	Yes
Female	32	Reading/computer screen	1–3	Yes	No	No	MA	20	3 days	Yes
Male	24	No	3–10	Yes	No	No	No	4	2 days	Yes
Female	43	Astigmatism	1–3	Yes	No	No	MA	32	3 weeks	No
Female	41	Short‐sighted	1–3	Yes	Yes	Yes	MA	27	2 weeks	Yes
Male	23	Short‐sighted	3–10	Yes	Yes	Yes	MA	6	6 days	Yes
Female	18	Concentration/looking at screens	<1	Yes	No	No	MA	5	1–2 weeks ago	No

*Note*: Demographic information can be seen in terms of age and sex of participants. “Attack frequency” refers to the number of headache attacks per month. “Other sensory aura” records whether participants experienced aura in other modalities, as well as visual aura. “Language aura” refers to aura where the participant experiences difficulty producing language. "Pro diagnosis” refers to whether or not the participant had a professional diagnosis. “Duration” refers to the number of years the individual has experienced migraine. “Last attack” refers to the time since the last attack (in days). “Visual trigger" refers to whether or not the individual finds that visual stimuli can trigger a migraine attack. One individual (shown in bold font) experienced MA without headache. “Sensory aura” refers to other sensory modalities aside from visual aura, as participants were included on the basis of experiencing visual aura.

Abbreviations: MA, migraine with aura; Na, not answered.

### SSVEP

**FIGURE 4:**
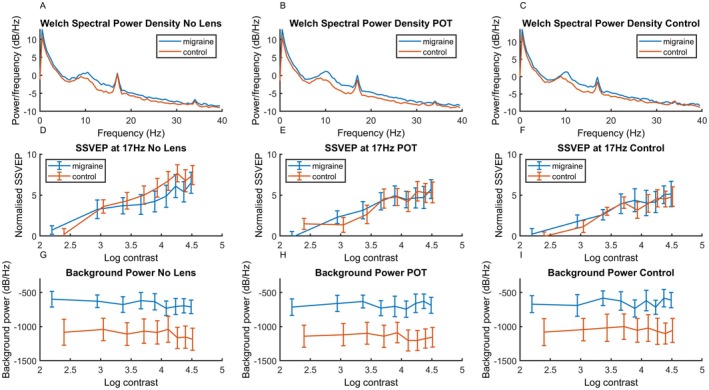
Results for experiment 1: top row (A–C) shows spectral power density functions for both MA and control groups for (left) no lens, (center) POT and (right) control lens conditions in response to 17 Hz flicker in Experiment 1. The response at the stimulation frequency 17 Hz can clearly be seen. Middle row (D–F) shows SSVEP against log contrast for migraine and control groups for (left) no lens, (center) POT, and (right) control lens conditions. There was an increase in SSVEP response with increasing contrast. Bottom row (G–I) shows background noise (average of surrounding frequencies) against log contrast for migraine and control groups for (left) no lens, (center) POT, and (right) control lens conditions. Background activity was higher for the MA compared to control group. Error bars indicate ±1 SE of the mean. Abbreviations: MA, migraine with aura; POT, precision ophthalmic tint; SE, standard error; SSVEP, steady‐state visual evoked potential. [Color figure can be viewed at wileyonlinelibrary.com]

The spectral density function can be seen in the top row of Figure 4A–C, showing a clear response to the stimulus frequency of 17 Hz. The SSVEP of the response for each level of contrast can be seen in the middle row of Figure 4D–F. Background activity level can be seen in the bottom row of Figure 4G–I.

The middle row of Figure 4D–F shows the SSVEP response for each of the nine contrast levels for the three lens conditions (no lens, POT, and control lens) for the 17‐Hz stimulation. A significant model including contrast, group and lens as fixed effects, transmission as a covariate, and observer as random effects emerged, accounting for 80% of the variance.

**FIGURE 5:**
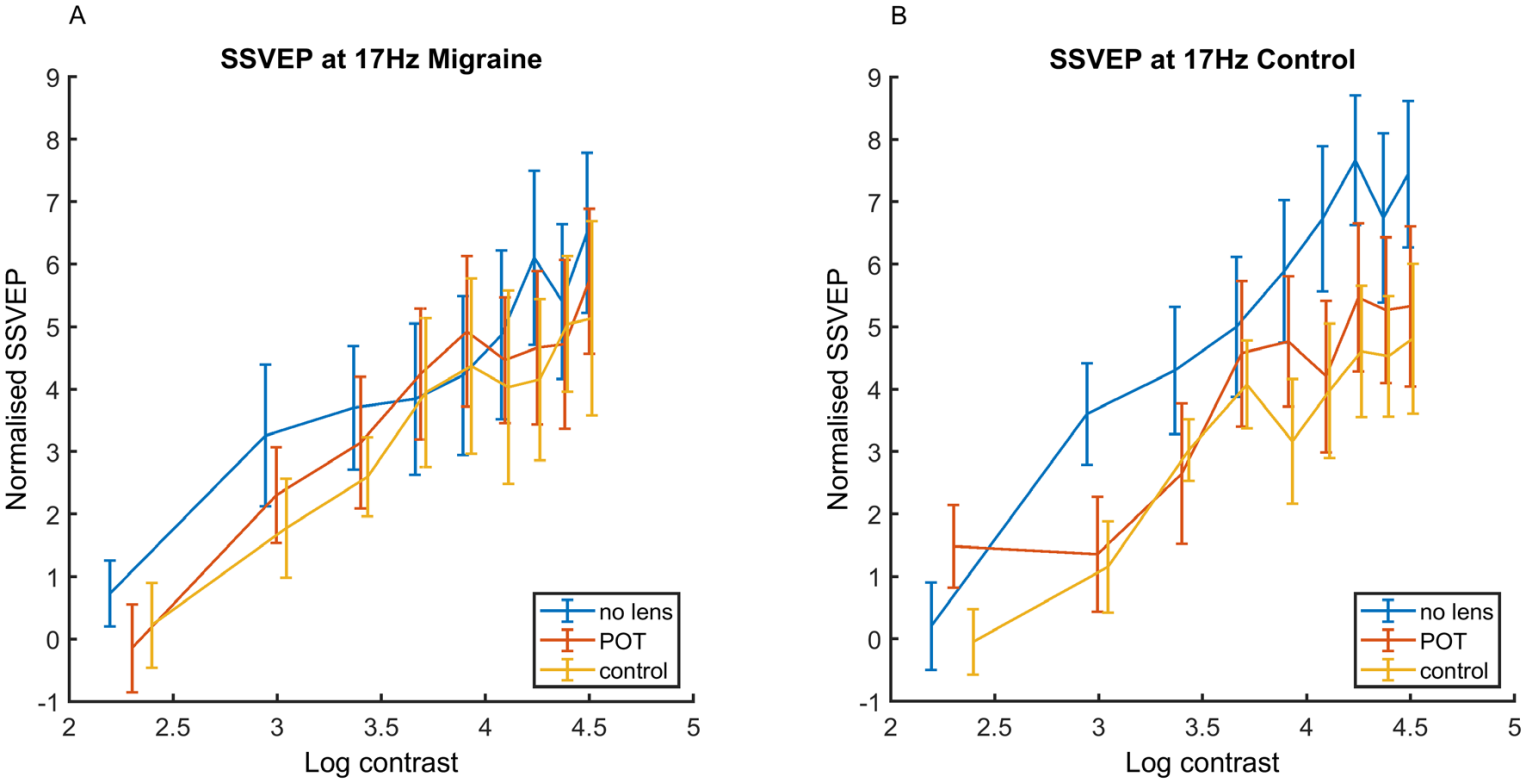
Results for experiment 1 showing the interaction effect: SSVEP in response to 17 Hz flicker against log contrast for no lens, POT and control lens conditions for (A, left) MA and (B, right) control groups. There is only a main effect of contrast for each of the three lens conditions. Error bars indicate ±1 SE of the mean. Abbreviations: MA, migraine with aura; POT, precision ophthalmic tint; SE, standard error; SSVEP, steady‐state visual evoked potential. [Color figure can be viewed at wileyonlinelibrary.com]

Results can be seen in Table 2. SSVEP increased with increasing contrast. There was an effect of transmission, and an effect of POT compared to no lens. There was a significantly lower increase in SSVEP with contrast for the POT and control lens compared to no lens. There was also a three‐way interaction between contrast, lens, and group. Figure 5 shows the SSVEP by contrast for the two groups separately, to show the effect of lens more clearly.Conducting a separate analysis for each lens condition separately revealed main effects of contrast only. These results can be seen in Table 3.

**TABLE 2:** Results for the SSVEP analysis using linear mixed effects model.

	Coefficient estimate	Lower CI	Upper CI	*p*‐value
Contrast	**3.21**	**2.34**	**4.09**	**<0.001**
Migraine	0.27	−1.54	2.08	0.768
POT	**1.91**	**0.10**	**3.71**	**0.038**
Control lens	1.71	−0.45	3.87	0.120
Transmission	**0.03**	**0.01**	**0.05**	**0.005**
Contrast × migraine	−0.89	−2.13	0.35	0.160
Contrast × POT	**−1.09**	**−1.81**	**−0.38**	**0.003**
Contrast × control lens	**−0.98**	**−1.69**	**−0.26**	**0.008**
Migraine × POT	−0.78	−2.67	1.12	0.422
Migraine × control lens	0.00	−1.99	1.99	0.998
Contrast × migraine × POT	**1.16**	**0.15**	**2.18**	**0.025**
Contrast × migraine × control lens	0.83	−0.18	1.85	0.108

*Note*: Migraine is compared to the control group as a reference. The no lens condition is used as the reference for comparison for the POT and control lenses. The lower and upper CIs represent lower and upper 95% CIs, respectively. Bold font indicates statistically significant results at *p* < 0.05.

Abbreviations: CI, confidence interval; POT, precision ophthalmic tint; SSVEP, steady‐state visual evoked potential.

**TABLE 3:** Results of the separate linear mixed effects models for SSVEP response for the no lens, POT, and control lens conditions in experiment 1.

	Coefficient estimate	Lower CI	Upper CI	*p*‐value
No lens				
Contrast	**3.21**	**2.34**	**4.09**	**<0.001**
Migraine	0.27	−1.56	2.11	0.771
Contrast × migraine	−0.89	−2.13	0.35	0.159
POT				
Contrast	**2.12**	**1.15**	**3.10**	**<0.001**
Migraine	−0.50	−2.12	1.12	0.542
Contrast × migraine	0.27	−1.11	1.65	0.699
Control lens				
Contrast	**2.24**	**1.25**	**3.23**	**<0.001**
Migraine	0.25	−1.06	1.56	0.704
Contrast × migraine	−0.02	−1.42	1.38	0.975

*Note*: Migraine with aura is compared to the control group as a reference. The lower and upper CIs represent lower and upper 95% CIs, respectively. Bold font indicates statistically significant results at *p* < 0.05.

Abbreviations: CI, confidence interval; POT, precision ophthalmic tint; SSVEP, steady‐state visual evoked potential.

### Background activity

Because there is increased background spectral power for MA compared to the control group, an additional analysis was conducted on the background level of the spectra. This consists of the sum of the power between 5 and 30 Hz, omitting 17 Hz. Figure 4 shows results for the baseline frequencies against log contrast on the bottom row.

A main effect model accounted for 92% of the variance, see Table 4. Importantly, there was greater overall background activity in the MA (mean = −666.45, SE = 116.67) compared to control group (mean = −1100.49, SE = 164.99) (estimated Cohen's *d* of 0.94; CI, 0.14–1.73). It should be noted that although there was a one‐sided heavy tailed distribution (see Supporting Information Part 2.2), linear mixed effects models are robust to these deviations from assumptions.^30^

**TABLE 4:** Results of the linear mixed effect model for background level of activity.

	Coefficient estimate	Lower CI	Upper CI	*p*‐value
Contrast	−16.47	−43.64	10.70	0.234
Migraine	**490.72**	**163.36**	**818.08**	**0.003**
POT	−61.07	−318.71	196.57	0.642
Control lens	−4.58	−344.22	335.06	0.979
Transmission	−0.51	−4.50	3.48	0.802

*Note*: The no lens condition is used as the reference for comparison for the POT and control lenses. The lower and upper CIs represent lower and upper 95% CIs, respectively. Bold font indicates statistically significant results at *p* < 0.05. Migraine is compared to the control group as a reference.

Abbreviations: CI, confidence interval; POT, precision ophthalmic tint; SSVEP, steady‐state visual evoked potential.

### Experiment 2

#### Methods

Experiment 2 was conducted to check whether the results were replicated in a new set of observers, and to elicit a stronger SSVEP response in both flicker frequencies. This was achieved by increasing the size of the stimulus to 8° and by using a square wave luminance profile (on–off) because this is predicted to elicit stronger SSVEP responses than sinusoidal variation in contrast.^32^, ^33^

#### Observers

Participants were recruited as in Experiment 1.

#### Apparatus

Apparatus are as Experiment 1.

#### Stimuli

Stimuli were similar to those in Experiment 1 but with some changes: the diameter was greater (8°), and the stimulation followed a square‐wave luminance profile (on–off).

#### Procedure

The procedure was the same as in Experiment 1 but with the addition of asking participants to rate the stimuli for discomfort after every presentation on a scale of 0–9, where 0 indicates no discomfort. Discomfort was not defined for observers, so as not to bias them to any particular interpretation. To reduce risk of confirmation bias (a tendency for observers to report in agreement), observers were encouraged to respond 0 if they experienced no discomfort. These data were analyzed with an ordinal regression model with group (MA or control), lens (no lens, POT, control lens), and flicker rate (5 and 17 Hz) as fixed effects, transmission as a covariate, and observer as a random effect (intercept). The generalized mixed effect models included group (MA or control), flicker rate (5 or 17 Hz), and lens (no lens, POT, and control lens) as fixed effects, and transmission of the lens as a covariate and observer as random effects.

### Analysis

In addition to the EEG analysis, an ordinal mixed effect model was fitted including group (MA or control), lens (no lens, POT, control lens), and flicker rate (5 and 17 Hz) as fixed effects, transmission as a covariate, and observer as a random effect using the package “ordinal” in R.^34^ Random intercept‐only model was used, because the random slope model failed to converge.

### Results

**FIGURE 6:**
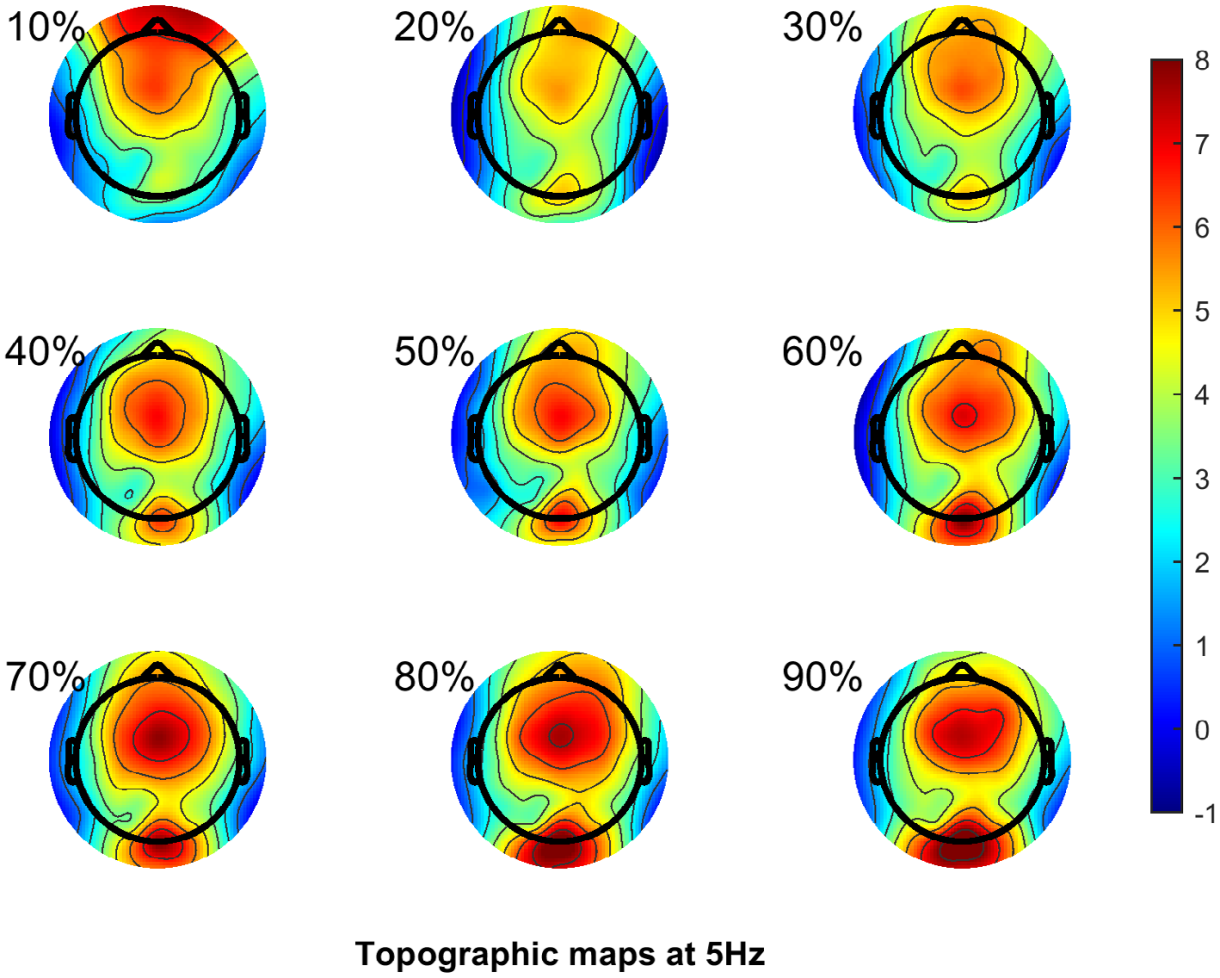
Scalp topography of electroencephalogram response to 5 Hz flicker in dB/Hz for experiment 2. Each individual scalp topography plot is for each individual level of contrast, 1 refers to 10% contrast through to 9 at 90% contrast. [Color figure can be viewed at wileyonlinelibrary.com]

Thirteen people with MA and 13 controls took part in experiment 2. The recording failed for one of the participants with MA, and one of the control participants was excluded because they experienced suspected tension‐type headache, leaving 12 per group to be included in the analysis. For the migraine group, the mean age was 30.1 ± 12.1 SD, 1 male, and for the control group the mean age was 24.3 ± 4.7 SD, 6 males. The migraine characteristics can be seen in Table 5. Figure 6 shows the scalp topography for the 5 Hz SSVEP. Compared to experiment 1, there is a stronger response in the occipital areas.

**TABLE 5:** Migraine group clinical characteristics.

Sex	Age	Glasses	Attack frequency (per month)	Moderate to severe pain	Other sensory aura	Language aura	Pro diagnosis	Duration (days)	Time since last attack	Visual trigger
Female	23	Short‐sighted	1–3	Yes	Yes	Yes	No	Na	1 week	Yes
Female	25	Reading/computer use	1–3	Yes	Yes	No	M	8	9 days	No
Female	55	Short‐sighted	<1	Yes	Yes	Yes	No	27	4 days	Yes
Female	40	Long‐sighted	<1	Yes	Yes	No	MA	7	8 days	Yes
Female	18	Reading	1–3	No	Yes	No	No	20	3 weeks	Yes
Female	20	None	1–3	Yes	No	No	MA	21	10 months	Yes
Female	25	Long‐sighted	<1	Yes	Yes	No	MA	34	2 months	No
Female	24	Short‐sighted and for migraine	1–3	Yes	Yes	No	MA	7	17 days	Yes
Female	45	Astigmatism	1–3	Yes	Yes	No	MA	14	10 months	Yes
Male	21	Short‐sighted	1–3	Yes	Yes	No	No	30	1 month	Yes
Female	42	Short‐sighted	1–3	Yes	Yes	Yes	Yes	8	2 days	Yes
Female	23	Short‐sighted	1–3	Yes	Yes	Yes	No	10	8 days	No

*Note*: Demographic information can be seen in terms of age and gender (biological sex) of participants. “Attack frequency” refers to the number of headache attacks per month. “Other sensory aura” records whether participants experienced aura in other modalities, as well as visual aura. “Language aura” refers to aura where the participant experiences difficulty producing language. “Pro diagnosis” refers to whether or not the participant had a professional diagnosis. “Duration” refers to the years the individual has experienced migraine (in years). “Last attack” refers to the time since the last attack (in days). “Visual trigger” refers to whether or not the individual finds that visual stimuli can trigger a migraine attack. “Sensory aura” refers to other sensory modalities aside from visual aura, as participants were included on the basis of experiencing visual aura.

Abbreviations: MA, migraine with aura; Na, not answered.

### SSVEP

**FIGURE 7:**
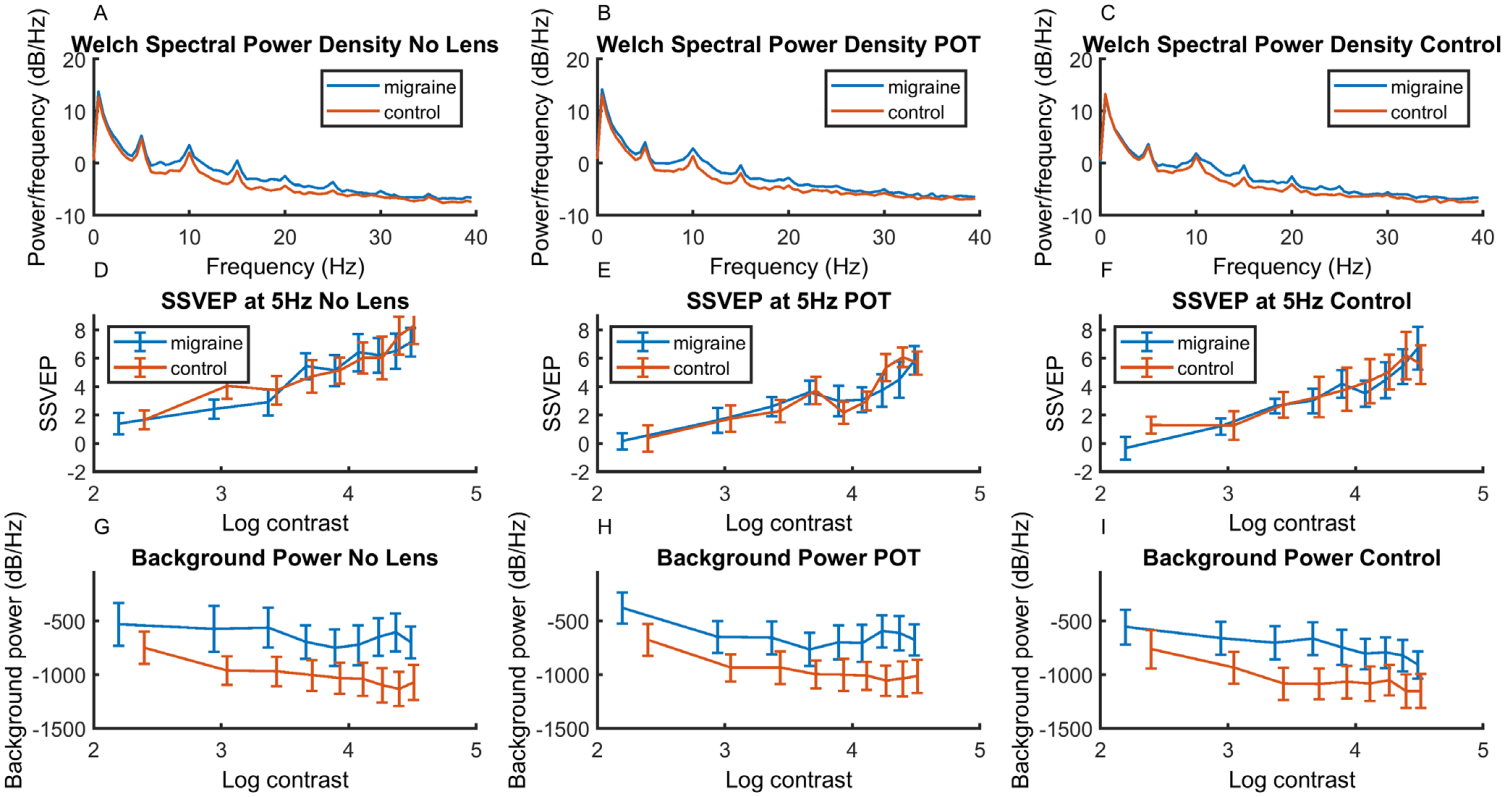
Results for experiment 2 at 5 Hz flicker frequency. Top row (A–C) shows spectral power density functions for both MA and control groups for (left) no lens, (center) POT, and (right) control lens conditions in response to 5 Hz flicker in experiment 2. This shows a SSVEP response at the stimulation frequency (5 Hz) and the harmonics. Middle row (D–F) shows SSVEP against log contrast for migraine and control groups for (left) no lens, (center) POT, and (right) control lens conditions. There was an effect of increasing SSVEP response with increasing contrast. Bottom row (G–I) shows background noise (average of surrounding frequencies) against log contrast for migraine and control groups for (left) no lens, (center) POT, and (right) control lens conditions. There was increased background activity with increased contrast. Error bars indicate ±1 SE of the mean. Abbreviations: MA, migraine with aura; POT, precision ophthalmic tint; SE, standard error; SSVEP, steady‐state visual evoked potential. [Color figure can be viewed at wileyonlinelibrary.com]

Figure 7 shows the results of experiment 2 for the 5 Hz flicker frequency. A statistically significant model emerged with main effects only, explaining 70% of the variance. There was no group difference between migraine and control groups in the amplitude of the SSVEP. SSVEP increases with increasing contrast. A complete set of results can be seen in Table 6.

**TABLE 6:** Results of the linear mixed effect model for SSVEP for 5 Hz stimulation frequency.

	Coefficient estimate	Lower CI	Upper CI	*p*‐value
Contrast	**2.48**	**1.92**	**3.05**	**<0.001**
Migraine	−0.52	−1.61	0.57	0.350
POT	−0.84	−3.23	1.56	0.494
Control lens	−0.30	−3.25	2.65	0.840
Transmission	0.02	−0.02	0.05	0.415

*Note*: Migraine is compared to the control group as a reference. The no lens condition is used as the reference for comparison for the POT and control lenses. The lower and upper CIs represent lower and upper 95% CIs, respectively. Bold font indicates statistically significant results at *p* < 0.05.

Abbreviations: CI, confidence interval; POT, precision ophthalmic tint; SSVEP, steady‐state visual evoked potential.

### Background activity

A statistically significant linear mixed effect model including only main effects emerged, explaining 90% of the variance. As contrast increased, the level of background activity at 5 Hz flicker decreased. There was a marginal effect of group, with a nonsignificant trend toward increased background activity in the migraine group (migraine group mean = −500.01, SE = 122.99, control group mean = −741.88, SE = 126.12, estimated Cohen's *d* = 0.72, CI, −0.13 to 1.58). For a full table of results, see Table 7.

**TABLE 7:** Results of the linear mixed effect model for background level of activity for 5 Hz stimulation.

	Coefficient estimate	Lower CI	Upper CI	*p*‐value
Contrast	**−122.47**	**−177.94**	**−66.99**	**<0.001**
Migraine	302.62	−55.42	660.65	0.097
POT	47.22	−327.02	421.46	0.804
Control lens	−38.29	−505.55	428.96	0.872
Transmission	0.31	−5.52	6.14	0.917

*Note*: Migraine is compared to the control group as a reference. The no lens condition is used as the reference for comparison for the POT and control lenses. The lower and upper CIs represent lower and upper 95% CIs, respectively. Bold font indicates statistically significant results at *p* < 0.05.

Abbreviations: CI, confidence interval; POT, precision ophthalmic tint.

### Experiment 2: 17 Hz

**FIGURE 8:**
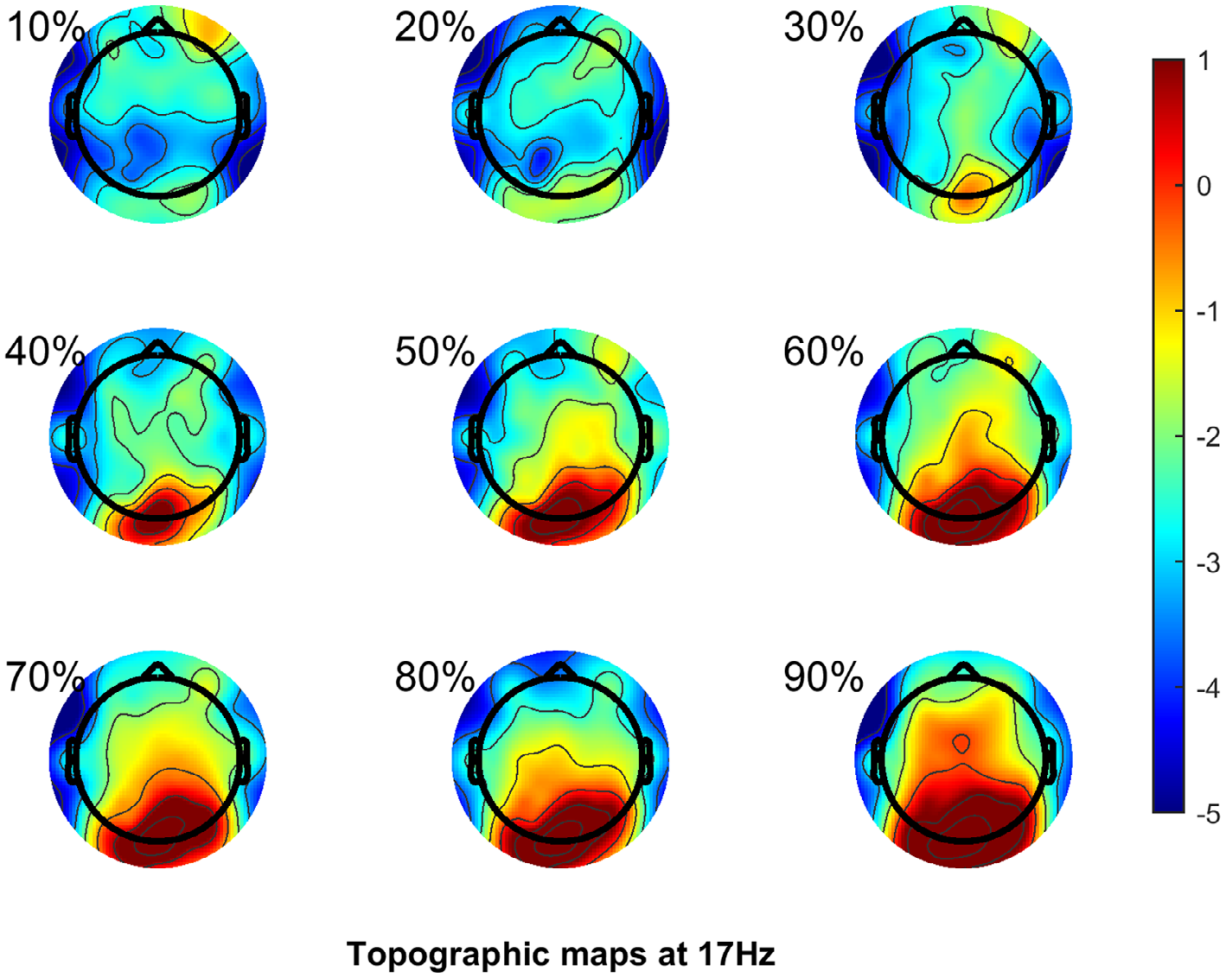
Scalp topography of electroencephalogram response to 17 Hz flicker in dB/Hz for experiment 2. Each individual scalp topography plot is for each individual level of contrast, 1 refers to 10% contrast, through to 9 at 90% contrast. [Color figure can be viewed at wileyonlinelibrary.com]

Figure 8 shows the scalp topography of the SSVEP response at 17 Hz.

**FIGURE 9:**
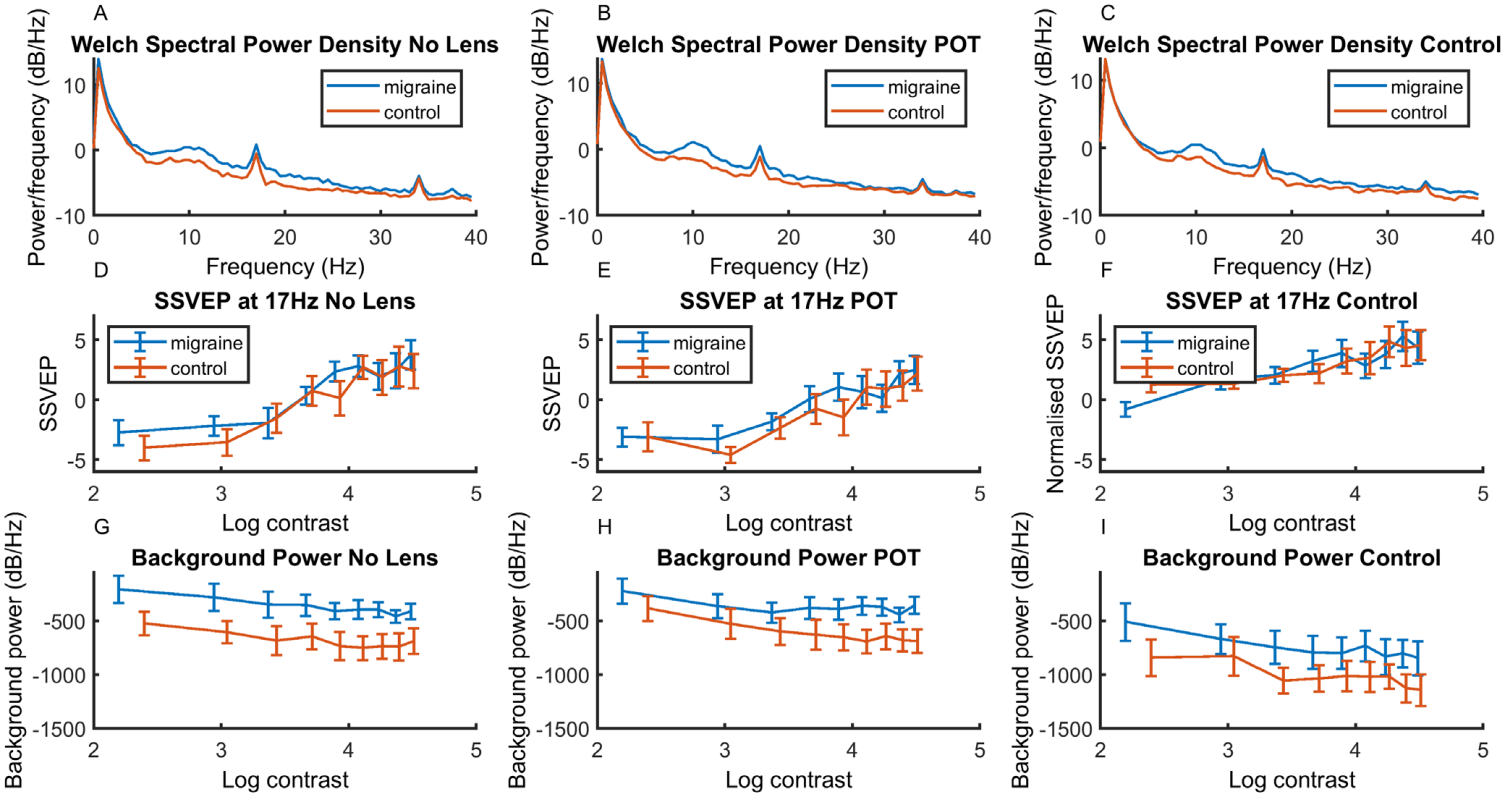
Results for experiment 2 at 17 Hz flicker frequency. Top row (A–C) shows spectral power density functions for both MA and control groups for (left) no lens, (center) POT, and (right) control lens conditions in response to 17 Hz flicker in Experiment 2. This shows a clear SSVEP response at the stimulation frequency (17 Hz) and harmonic. Middle row (D–F) shows SSVEP against log contrast for migraine and control groups for (left) no lens, (center) POT, and (right) control lens conditions. SSVEP response increased with increasing contrast. Bottom row (G–I) shows background noise (average of surrounding frequencies) against log contrast for migraine and control groups for (left) no lens, (center) POT, and (right) control lens conditions. There was greater background activity for the MA group compared to the control group. Error bars indicate ±1 SE of the mean. Abbreviations: MA, migraine with aura; POT, precision ophthalmic tint; SE, standard error; SSVEP, steady‐state visual evoked potential. [Color figure can be viewed at wileyonlinelibrary.com]

Figure 9 shows the results of experiment 2 for the 17 Hz flicker frequency.

### Experiment 2: 17 Hz SSVEP

**FIGURE 10:**
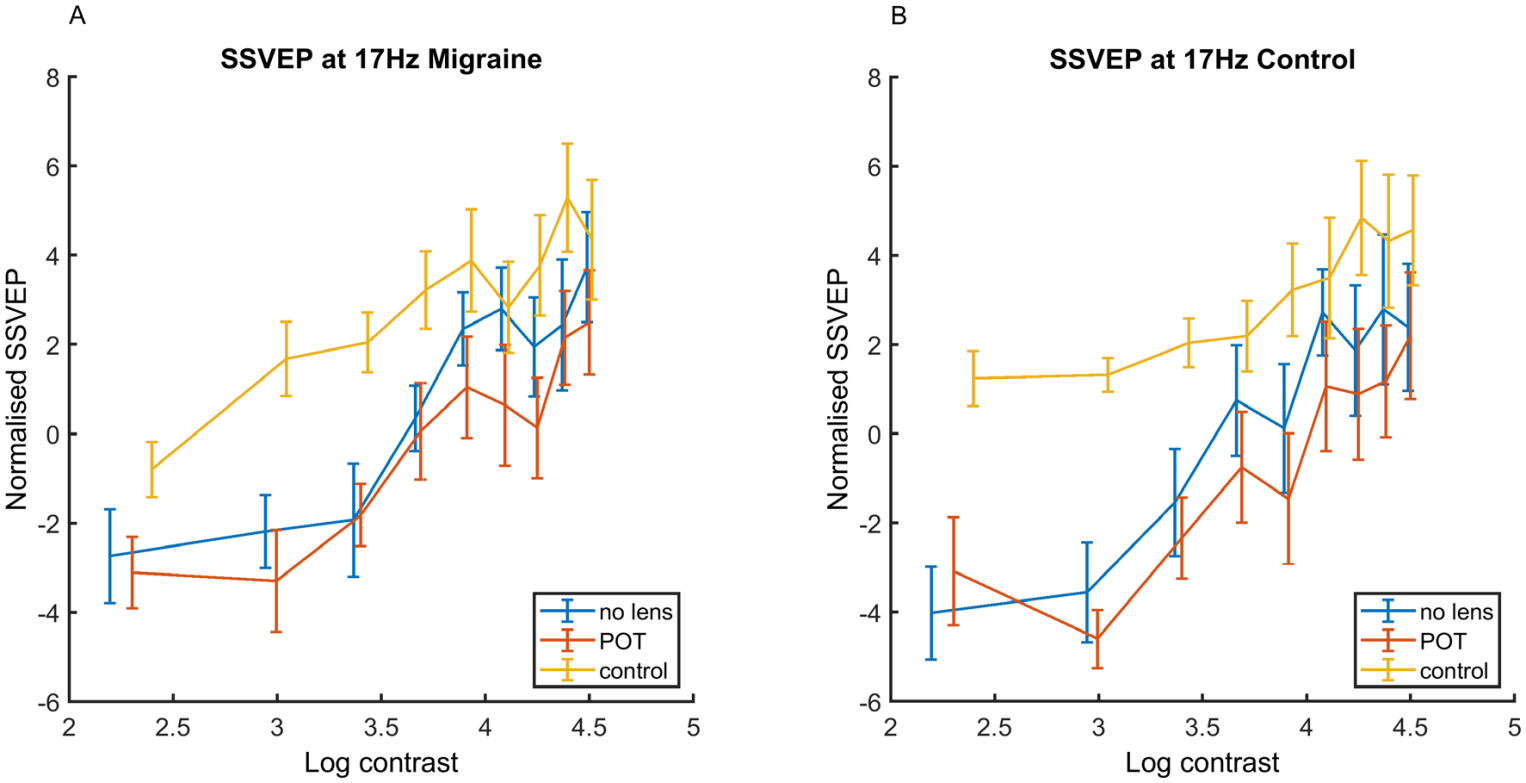
Results for experiment 2 at 17 Hz flicker frequency showing the interaction more clearly. SSVEP against log contrast for no lens, POT, and control lens conditions for (A, left) MA and (B, right) control groups for 17 Hz stimulation in experiment 2. The overall response is higher, but the contrast response is flatter, for the control lens compared to no lens condition. Error bars indicate ±1 SE of the mean. Abbreviations: MA, migraine with aura; POT, precision ophthalmic tint; SE, standard error; SSVEP, steady‐state visual evoked potential. [Color figure can be viewed at wileyonlinelibrary.com]

A significant interaction model emerged, accounting for 72% of the variance. There was an effect of increasing contrast on the SSVEP. In addition, there was an increase in SSVEP amplitude with the control lens compared to the no lens condition, but the effect of contrast was reduced for the control lens compared to no lens. The full set of results can be seen in Table 8 and in Figure 10.

**TABLE 8:** Results of the linear mixed effect model for SSVEP at 17 Hz flicker.

	Coefficient estimate	Lower CI	Upper CI	*p*‐value
Contrast	**3.50**	**2.18**	**4.81**	**<0.001**
Migraine	1.06	−1.14	3.25	0.345
POT	−0.74	−3.44	1.96	0.591
Control lens	**4.28**	**1.07**	**7.49**	**0.009**
Transmission	−0.01	−0.05	0.02	0.463
Contrast × migraine	−0.33	−2.20	1.53	0.726
Contrast × POT	−0.70	−1.66	0.26	0.154
Contrast × control lens	**−1.73**	**−2.68**	**−0.77**	**<0.001**
Migraine × POT	−0.32	−2.68	2.05	0.793
Migraine × control lens	−1.93	−4.26	0.41	0.106
Contrast × migraine × POT	0.25	−1.10	1.61	0.712
Contrast × migraine × control lens	0.91	−0.45	2.27	0.188

*Note*: Migraine is compared to the control group as a reference. The no lens condition is used as the reference for comparison for the POT and control lenses. The lower and upper CIs represent lower and upper 95% CIs, respectively. Bold font indicates statistically significant results at *p* < 0.05.

Abbreviations: CI, confidence interval; POT, precision ophthalmic tint; SSVEP, steady‐state visual evoked potential.

### Background activity

A significant main effect model emerged accounting for 89% of the variance. There was a reduction in background activity with increasing contrast. Importantly, there was again increased background noise in the migraine compared to the control group, estimated Cohen's *d* of 0.95, CI, 0.05–1.84. There was also an effect of control lens compared to the no lens condition. A complete set of results can be seen in Table 9.

**TABLE 9:** Results of the linear mixed effect model for background level of activity for 17 Hz flicker.

	Coefficient estimate	Lower CI	Upper CI	*p*‐value
Contrast	**−106.99**	**−151.34**	**−62.65**	**<0.001**
Migraine	**292.83**	**55.04**	**530.62**	**0.016**
POT	−68.33	−414.45	277.79	0.698
Control lens	**−479.25**	**−924.96**	**−33.54**	**0.035**
Transmission	−1.71	−7.24	3.82	0.544

*Note*: Migraine is compared to the control group as a reference. The no lens condition is used as the reference for comparison for the POT and control lenses. The lower and upper CIs represent lower and upper 95% CIs, respectively. Bold font indicates statistically significant results at *p* < 0.05.

Abbreviations: CI, confidence interval; POT, precision ophthalmic tint.

### Behavioral results

**FIGURE 11:**
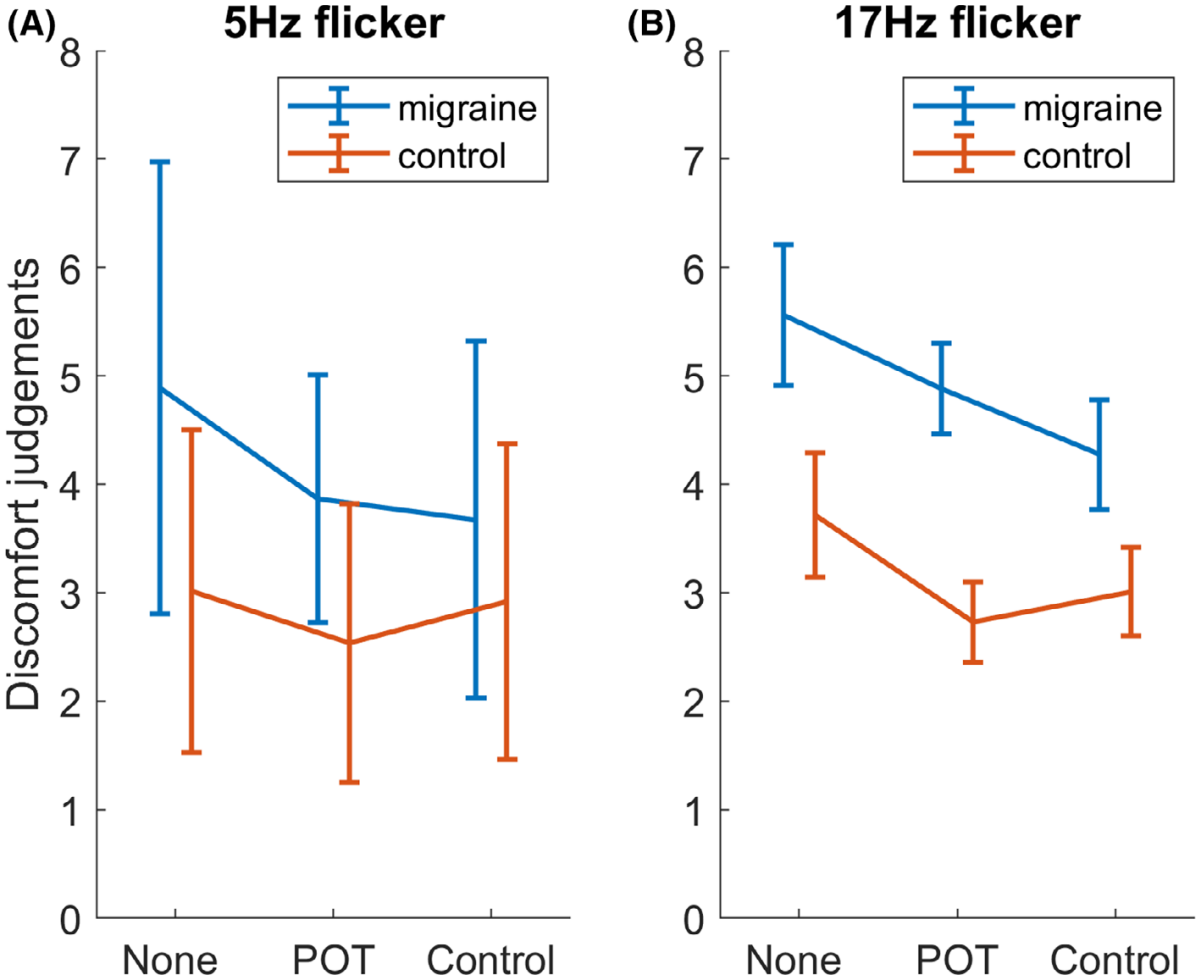
Behavioral results of the experiment 2. Average discomfort judgments are plotted against lens condition for migraine with aura and control groups. (A) In response to 5 Hz flicker. (B) Responses to 17 Hz flicker. Error bars are ±1 SE of the mean. Abbreviation: SE, standard error. [Color figure can be viewed at wileyonlinelibrary.com]

Figure 11 shows the discomfort judgments for the MA and control groups for both 5 and 17 Hz flicker.

Results of the ordinal mixed effect model can be seen in Table 10. Discomfort judgments were higher for the MA compared to the control group (median for control group = 3, IQR = 3, median for migraine group = 4, IQR = 4). 17 Hz flicker was more uncomfortable overall compared to 5 Hz flicker (median for 5 Hz = 3, IQR = 3, median for 17 Hz = 4, IQR = 4). There was an effect of transmission. Discomfort judgments were reduced with the POT and the control lens compared to no lens. The effect of group was smaller for both the POT and the control lens conditions.

**TABLE 10:** Results of the generalized mixed effect model for behavioral responses.

	Coefficient estimate	Lower CI	Upper CI	*p*‐value
POT	**1.66**	**0.65**	**2.674**	**0.001**
Control lens	**1.83**	**0.61**	**3.05**	**0.003**
Flicker	**0.97**	**0.49**	**1.45**	**<0.001**
Migraine	**3.58**	**1.18**	**6.00**	**0.003**
Transmission	**0.03**	**0.01**	**0.04**	**<0.001**
POT × flicker	−0.57	−1.27	0.13	0.109
Control × flicker	−0.67	−1.36	0.036	0.059
Migraine × POT	**−1.09**	**−1.77**	**−0.40**	**0.002**
Migraine × control lens	**−1.18**	**−1.86**	**−0.50**	**<0.001**
Migraine × flicker	−0.13	−0.80	53.96	0.702
Migraine × flicker × POT	0.75	−0.21	1.71	0.124
Migraine × flicker × control lens	0.58	−0.38	1.546	0.238

*Note*: The migraine group is compared to the control group as a reference. The no lens condition is used as the reference for comparison for the POT and control lenses. The 5 Hz flicker rate is compared to the 17 Hz flicker rate as a reference. The lower and upper CIs represent lower and upper 95% CIs, respectively. Bold font indicates statistically significant results at *p* < 0.05.

Abbreviations: CI, confidence interval; POT, precision ophthalmic tint.

## DISCUSSION

The main objective in the current study was to investigate whether MA showed a similar reduction in contrast gain control at high contrasts, as found in photosensitive epilepsy.^20^ In the current study, we did not show evidence of a reduced contrast gain control in MA. Porciatti et al.^20^ showed differences between groups at the slower temporal frequencies tested. These data were not available for experiment 1, and no group differences of this kind were evident in experiment 2. Overall, there was therefore no evidence of reduced contrast gain control in MA.

In both experiments, when presented with 17 Hz stimulation, individuals with migraine showed an increased level of background EEG activity (activity unrelated to the stimulus) but relatively small differences in the amplitude of the evoked response. This is consistent with increased additive neural noise (noise that does not scale with the stimulus) in MA, an idea previously suggested to reconcile high amplitude evoked potentials and poorer performance on visual tasks.^18^ Other authors have suggested an increase in internal noise in migraine, possibly due to increased neural variability, based on behavioral methods.^19^, ^35^, ^36^ In the current study, the increase in background activity in the migraine group was constant across stimulus contrast levels, which indicates that it does not scale with the stimulus; therefore, this is an increase in additive (does not scale with the stimulus), rather than multiplicative noise (scales with the stimulus).

A second objective in this study was to investigate whether there were any effects of POTs on the SSVEP response in MA, as there are several reports of benefits of lenses on headaches.^37^, ^38^ There are also reports of effects in MA when using POTs compared to control lenses for visual search performance,^39^ and on the hemodynamic response magnitude.^11^ In experiment 1 of the current study, the effect of contrast on the SSVEP response was reduced when using either the POT or control lens. Both lenses will reduce the overall signal, and possibly visibility of the signal. However, in experiment 2 at 17 Hz stimulation, there was an increase in SSVEP with the control lens compared to the no lens condition, rather than a reduction. Transmission was included as a covariate, which should have mitigated the possibility that effects are due to reduced transmission. Previous authors showed effects of POTs on basic tasks of visual function, but this was an *increase* in contrast discrimination thresholds, representing poorer performance.^40^ This might support the notion that although lenses might be more comfortable, clarity might be reduced.

In the current study, there was no interaction effect between lens and migraine group, suggesting that any effect of lens is independent of migraine status. In addition, in previous work, people with MA have chosen more saturated colors compared to controls,^39^, ^40^ but this was not the case in the current study. One possible explanation for the lack of an interaction effect might therefore be that our participants did not experience high levels of visual discomfort in comparison with previous research, but we do not have the data on visual discomfort from the prior work to enable this comparison. Alternatively, prior fMRI research that has found differential effects of POTs in migraine and has shown these differences in higher visual areas (V3 and V4) rather than V1. Because the current SSVEP paradigm is biased toward responses in early visual areas, it may be that other methods may detect processing differences at later processing stages.

In previous work,^34^, ^35^ people with MA tended to choose more saturated colors compared to controls. In the current experiment, people with MA did not chose systematically more saturated colors compared to controls. SSVEP analysis was also conducted comparing the group that chose the more saturated colors compared to those with choice closer to the daylight locus (less saturated colors). The reason for this is two‐fold; first, there are differences in transmission of the lenses, and second, there is the possibility of systematic differences between high and low saturation groups, independent of migraine status. One possibility is susceptibility to visual discomfort; although this is commonly higher in MA, it is also possible for people without MA to experience visual discomfort.^41^ This additional analysis can be seen in the Supporting Information Part 4 and Part 5.3.

The MA group showed greater discomfort compared to the control group, and the 17 Hz flicker was judged as more uncomfortable than the 5 Hz flicker. It has been suggested that visual discomfort can arise from excessive responses to visual stimuli as a result of inefficient processing.^42^, ^43^ It is possible that increased noise in migraine leads to increased discomfort from visual stimuli as neural noise might mean that images are less efficiently processed in people with MA compared to other people.^18^ As well as the effect of flicker, there was also an effect of lens. Compared to no lens, both the POT and the control lens reduced discomfort. Because there was also an effect of transmission, this may imply that any reduction in stimulus luminance (in this case through filtering) reduces the discomfort. There is a possible benefit from color, although in this sample, this color did not need to be precise because there was a benefit for both the POT and the control lens.

There are some limitations to the study. All participants were recruited from a single location and so generalizability may be reduced. Participant classification was based on self‐reported fulfillment of the International Headache Society classification criteria. There was no consultant neurologist associated with this study. However, many in the sample did additionally have a professional diagnosis of migraine (see Tables 1 and 5). This is representative because only approximately 64% people with migraine seek a professional diagnosis.^44^ Estimates of attention and fatigue were not measured in this study. However, this was mitigated due to the study being relatively short (using the SSVEP method), and participants took regular breaks to limit exposure. In addition, conditions were counterbalanced and this will have mitigated effects of attention and fatigue on the SSVEP responses.

The response to 5‐Hz stimulation in experiment 1 was too weak to appear against the background levels of activity, either for the MA or the control group. SSVEPs for 5‐Hz stimulation have been measured previously;^20^, ^45^ however, the size of the visual stimulus in the previous studies was much larger than in the current study—stimuli subtended 21 × 17°^45^ and 14°.^20^ For ethical reasons, this stimulus was kept small in experiment 1—there are reports that visual stimuli can trigger headache.^4^, ^46^ No symptoms were reported and so experiment 2 was designed with a stronger stimulus to elicit a more pronounced SSVEP effect.

The measures in the current study are electrophysiological, aimed at addressing the theoretical mechanism of MA. Neural noise has been suggested to account for the reported differences in behavioral performance in people with migraine compared to controls.^18^ Increased neural noise has also been suggested to be a contributing factor to cortical spreading depression.^1^, ^47^ In the current study, we find evidence to support the suggestion that increased neural noise may be a contributing factor in MA. Translating these electrophysiological findings into predictions for magnitude of any behavioral effects of noise is an avenue for future research.

## CONCLUSION

There was no evidence of reduced gain control in MA, but instead there was evidence of increased additive background activity. This increased background activity may be related to neural activity that is unrelated to the stimulus, which is increased noise. This provides a simple account for the increased electrophysiological responses, yet poorer performance on perceptual tasks shown in previous research. In the current study, neural activity was inferred from electrophysiological responses measured using EEG. There is some evidence that colored spectacles—POTs or the control lens—were judged to make aversive stimuli more comfortable in comparison to no lens. However, this does not seem to relate to electrophysiological responses from early visual areas. It remains for future research to investigate how POTs might relate to functional significance; one possibility is that the mechanism is in a later processing stage in the brain.

## AUTHOR CONTRIBUTIONS

**Louise O'Hare:** Conceptualization; data curation; formal analysis; investigation; visualization; writing – original draft; writing – review and editing. **Paul B. Hibbard:** Conceptualization; methodology; writing – original draft; writing – review and editing. **Arnold J. Wilkins:** Conceptualization; methodology; writing – original draft; writing – review and editing.

## FUNDING INFORMATION

There was no specific external funding associated with this research.

## CONFLICT OF INTEREST STATEMENT

**Arnold J. Wilkins** received royalties from the Intuitive Colorimeter Mark 4 (Curve) that were donated to the University of Essex for student support. **Louise O'Hare** and **Paul B. Hibbard** declare no conflicts of interest.

## Supporting information


Data S1.

## Data Availability

All data for this study, the code to display the stimuli, and analysis scripts are freely available at the Open Science Framework (https://osf.io/3w89b/).
