# Supplementary material for: Evidence for increased background neural noise in migraine with aura: Hyperactive but not hyperresponsive
Source: Headache. 2025 Sep 16;66(3):615–32. doi: 10.1111/head.15046 (PMC12951707; doi:10.1111/head.15046)
Supplement: Supplementary file 1 — Data S1. [file HEAD-66-615-s001.docx]

Supplementary Information

**1) Results for Experiment 1 (5Hz flicker)**

In Experiment 1, 5Hz stimulation did not result in measurable SSVEP responses, possibly due to the size of the stimulus. Scalp topography of stimulation at 5Hz are shown in Supplementary Figure 1.


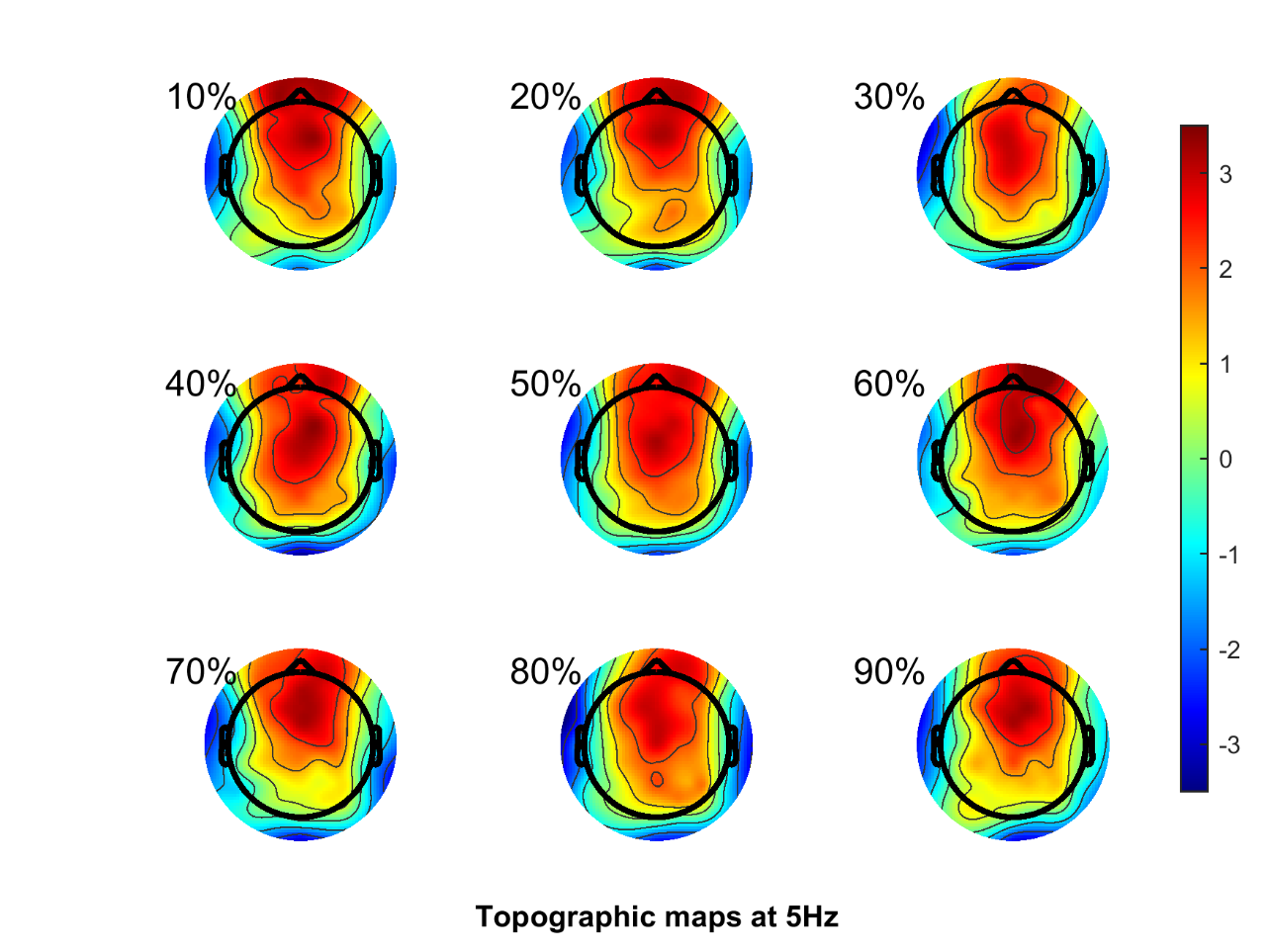


*Supplementary Figure 1: Scalp topography of EEG response to 5Hz flicker, in dB/Hz. Each individual scalp topography plot is for each individual level of contrast, 1 refers to 10% contrast, through to 9 at 90% contrast.*

The spectral power density function (Supplementary Figure 2) shows no peak at 5Hz in response to the stimulus. As with the 17Hz stimulation, the SSVEP for the 5Hz stimulus was estimated by subtracting the average of the surrounding 1Hz, in this case 4-6 Hz. The lower row shows SSVEP response against log contrast. There is no increase in SSVEP response with increasing stimulus contrast, confirming that the response was too weak to measure for this flicker frequency.


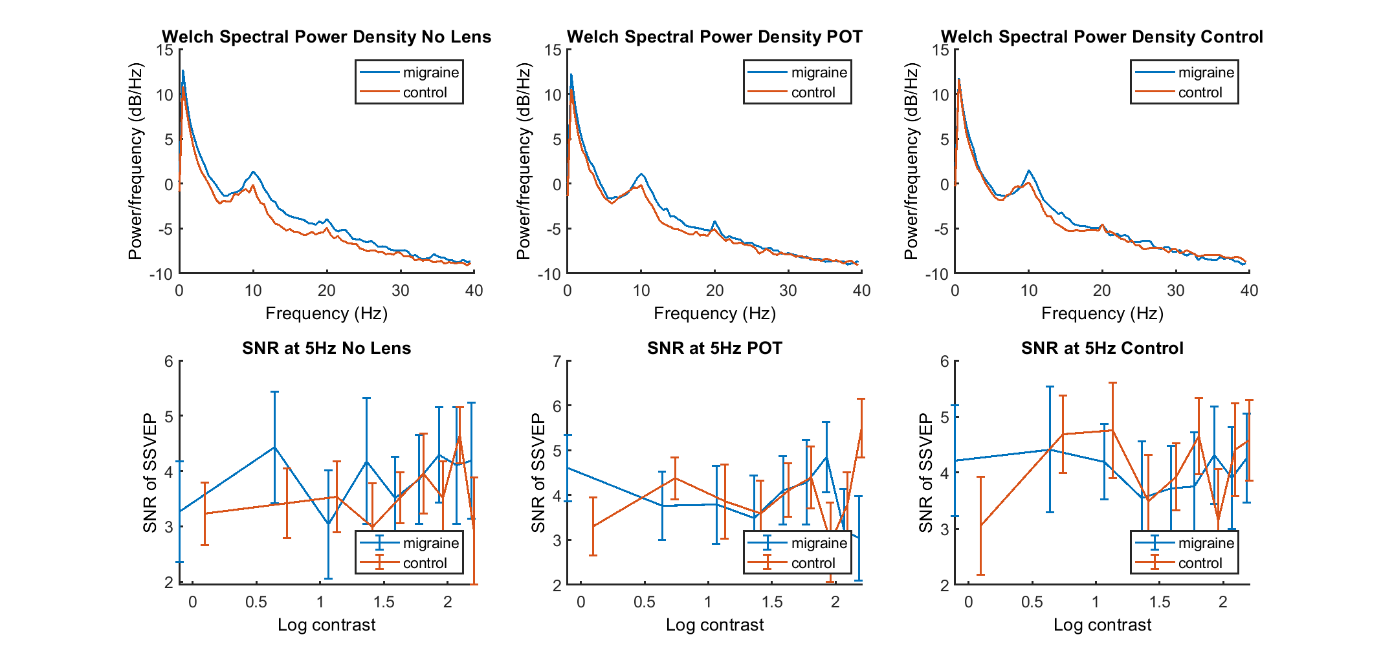


*Supplementary Figure 2: Top row shows spectral power density of the response at 5Hz stimulation for the migraine and control groups for (left) no lens, (centre) POT and (right) control lens conditions. There is no apparent peak in the response at 5Hz. Bottom row shows the SSVEP for the migraine and control groups against log contrast for (left) no lens, (centre) POT and (right) control lens conditions. Error bars indicate ±SE of the mean.*

**2) Model Selection and Assumptions of the Linear Mixed Effect Models (Experiment 1)**

2.1) Experiment 1: 17Hz SSVEP

Assumptions of the linear mixed effect models used for statistical inference were established via visual inspection. Supplementary Figure 3 shows that the distribution SSVEP responses is relatively normal with a moderate skew of 0.60. A model with a main effect and interactions was included as this gave a better fit (as measured by the log ratio test) than either a null model or one with only main effects (Supplementary Table 1). In addition, models including random slopes of contrast and lens were found to give a better fit compared to a model with random intercept only. Again, the decision criterion was to compare the log-likelihood of the two models, with and without random slopes.

Assumptions for the distributions of residuals were also tested via visual inspection (Supplementary Figure 4). The top figure shows the probability of residuals following a normal distribution, represented by a dotted line. The residuals are close to normal. The lower plot shows the scatterplot of the residuals against the fitted values, showing no evidence of any patterns in the residuals.


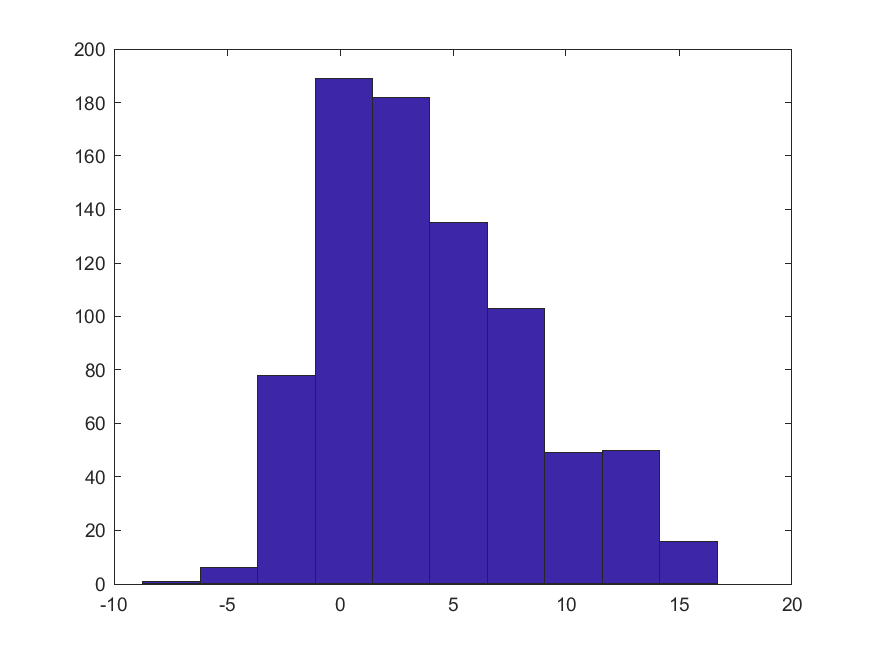


*Supplementary Figure 3: Histogram of the SSVEP response data at 17Hz.*

*Supplementary Table 1: Model build table, showing comparison to simpler models. RE = random effects, FE = fixed effects, LRT = simulated likelihood ratio test, AIC = Akaike Information Criterion, BIC = Bayes Information Criterion, LL = log-likelihood, df = degrees of freedom. All models are statistically significantly different from the null model.*

| Model specification | Model name | Nested /simpler model | Fixed effects added |  | Model Fit | | | | LRT Test | | |
| --- | --- | --- | --- | --- | --- | --- | --- | --- | --- | --- | --- |
|  | | | |  | AIC | BIC | LL | df | df | Χ^2^ | p |
| RE only | Null | - | - |  | 4171.5 | 4190.3 | -2081.8 | 4 |  |  |  |
| **FE main effects** | **Main effects** | **Null** | **Contrast + lens + group** |  | **3884.7** | **3922.3** | **-1934.4** | **8** | **4** | **294.8** | **<0.001** |
| **Interactions** | **Interactions** | **Main effects** | **Contrast * lens * group** |  | **3881.7** | **3952.2** | **-1925.9** | **15** | **7** | **17.0** | **0.02** |
| **Interactions (random slope contrast)** | **Interactions** | **Interactions (intercept only)** | **Contrast * lens * group** |  | **3759.4** | **3839.2** | **-1862.7** | **17** |  | **126.3** | **<0.001** |
| **Interactions (random slope contrast and lens)** | **Interactions** | **Interactions (intercept only)** | **Contrast * lens * group** |  | **3701.7** | **3809.7** | **-1827.8** | **23** |  | **196.1** | **<0.001** |


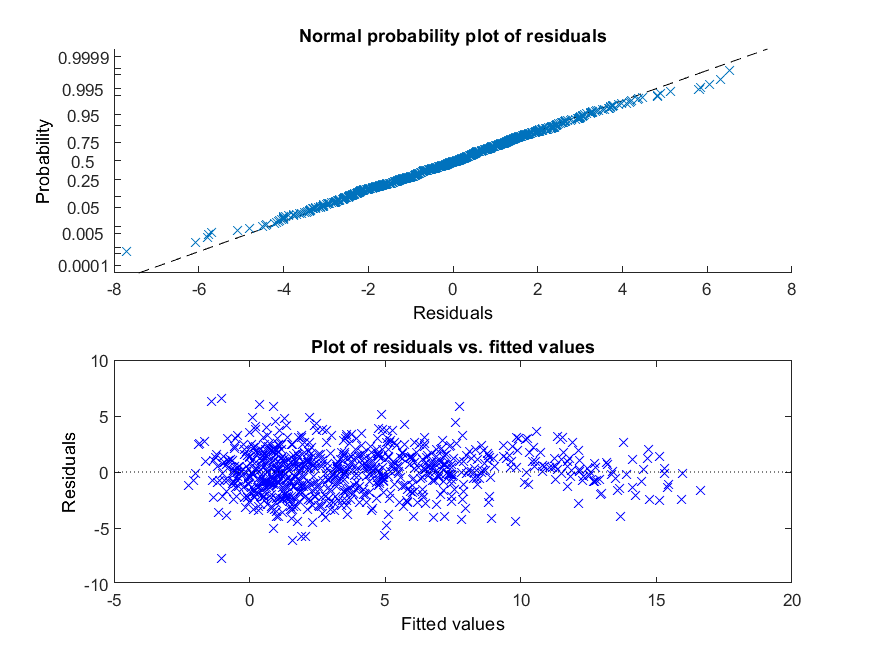


*Supplementary Figure 4: Top shows the probability of normal residuals from the linear mixed effects model of the SSVEP (normal distribution represented by the dotted line), bottom shows residuals against fitted values from the linear mixed effects model of the SSVEP.*

2.2) Experiment 1: Background Activity

Supplementary Figure 5 shows the distribution of the background activity data (total activity over trials between 6 and 30Hz, omitting 17Hz) to be close to normal, with a skewness of -0.16. The model selection process can be seen in Supplementary Table 2. A model including only main fixed effects of contrast, lens and group, and the random effect of observer and random effect of transmission was fitted was used, as including interaction terms did not improve the goodness of fit (log ratio test). Including random intercept and random slopes of contrast and lens gave a better fitting model compared to an intercept only model.

Supplementary Figure 6, top figure, shows the probability of residuals following a normal distribution, represented by a dotted line. This shows a slight deviation from normal, with some heavy tails at one end. The lower plot shows the scatterplot of the residuals against the fitted values.


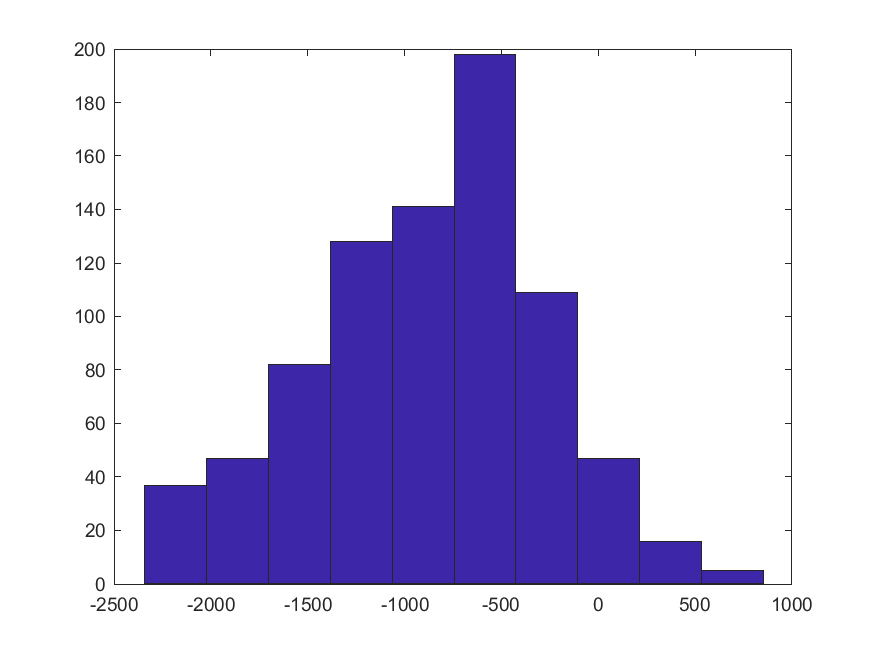


*Supplementary Figure 5: Histogram of the background activity, total over 6 to 30Hz, omitting 17Hz as the stimulation frequency.*

*Supplementary Table 2: Model build table, showing comparison to simpler models. RE = random effects, FE = fixed effects, LRT = simulated likelihood ratio test, AIC = Akaike Information Criterion, BIC = Bayes Information Criterion, LL = log-likelihood, df = degrees of freedom. All models are statistically significantly different from the null model.*

| Model specification | Model name | Nested /simpler model | Fixed effects added |  | Model Fit | | | | LRT Test | | |
| --- | --- | --- | --- | --- | --- | --- | --- | --- | --- | --- | --- |
|  | | | |  | AIC | BIC | LL | df | df | Χ^2^ | p |
| RE only | Null | - | - |  | 11447 | 11466 | -5719.6 | 4 |  |  |  |
| **FE main effects** | **Main effects** | **Null** | **Contrast + lens + group** |  | **11440** | **11477** | **-5711.9** | **8** | **4** | **15.4** | **<0.001** |
| Interactions | Interactions | Main effects | Contrast * lens * group |  | 11448 | 11519 | -5709.1 | 15 | 7 | 5.6 | 0.585 |
| FE main effects (random slope contrast) | Main effects | Main effects (interaction only) | Contrast + lens + group |  | 11440.0 | 11477.0 | -5711.9 | 10 | 2 | 2.5 | 0.290 |
| **FE main effects (random slope contrast and lens)** | **Main effects** | **Main effects (interaction only)** | **Contrast + lens + group** |  | **11441.0** | **11488.0** | **-5710.7** | **16** | **8** | **371.2** | **<0.001** |


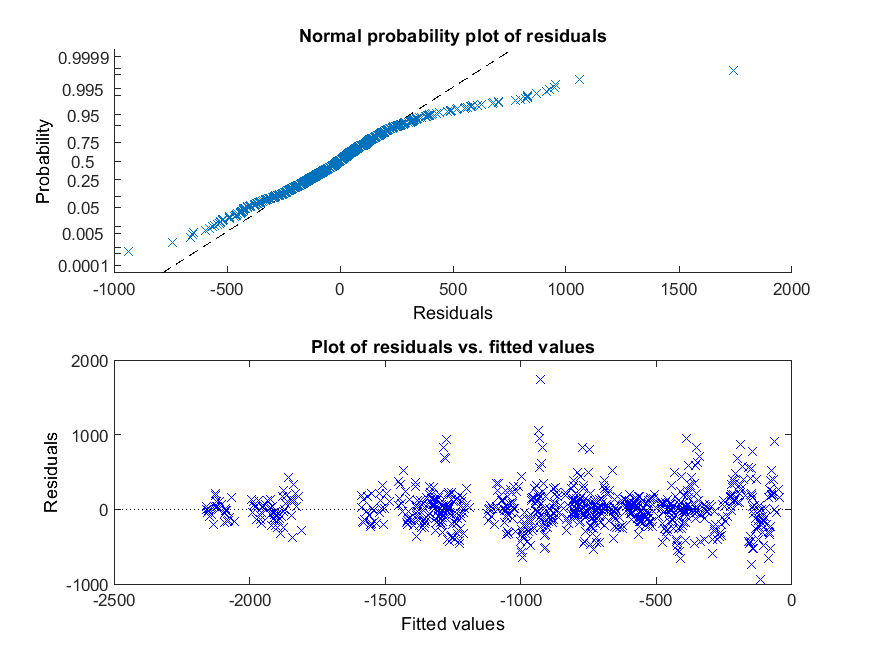


*Supplementary Figure 6: Top shows the probability of normal residuals from the linear mixed effects model for the background noise (normal distribution represented by the dotted line), bottom shows residuals against fitted values from the linear mixed effects model of the background noise.*

**3) Experiment 1 – omitting the observer with aura without headache**

In Experiment 1, one of the MA observers did not experience headache, but instead experienced aura without headache. The following shows a re-analysis of the Experiment 1 results without including the observer.

Supplementary Figure 7 shows the results for the analysis omitting the observer who experienced migraine aura without headache. The top row shows the spectra, the middle row the SSVEP response, and the bottom row the background activity.


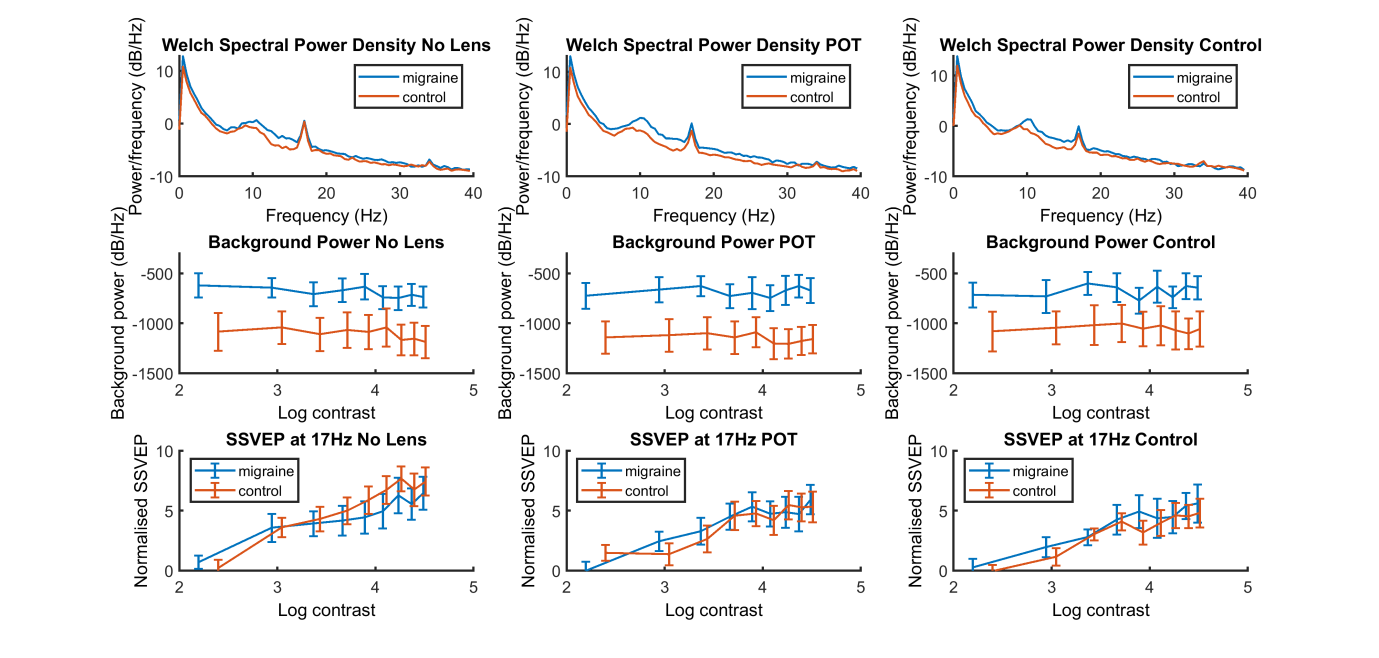


*Supplementary Figure 7: Top row shows spectral power density of the response at 17Hz stimulation for the migraine and control groups for (left) no lens, (centre) POT and (right) control lens conditions. This is without the observer who experienced aura without headache. Bottom row shows the SSVEP for the migraine and control groups against log contrast for (left) no lens, (centre) POT and (right) control lens conditions. Error bars indicate ±SE of the mean.*

A linear mixed effects model was fitted to predict SSVEP responses including group, lens and contrast as fixed effects and observer as a random effect (intercept and slope of contrast and lens), and their interactions. A significant interaction model emerged accounting for 80% of the variance.

There was a significant effect of increasing SSVEP with increasing contrast. There was a main effect of POT, and of control lens. There was also an interaction between migraine and contrast, the MA group showed a flatter SSVEP response to stimuli of increasing contrast. There was also an interaction between contrast and lens, specifically, there is a reduced slope for the SSVEP response to contrast with either the POT lens, or the control lens, compared to no lens. The full set of results can be seen in Supplementary Table 3.

*Supplementary Table 3: Results for the SSVEP analysis using linear mixed effects model, omitting the observer who experienced migraine aura without headache. Migraine is compared to the control group as a reference. The no lens condition is used as the reference for comparison for the POT and control lenses.*

|  | Coefficient estimate | Lower CI | Upper CI | p-value |
| --- | --- | --- | --- | --- |
| **Contrast** | **3.21** | **2.32** | **4.10** | **<0.001** |
| Migraine | 0.44 | -1.42 | 2.30 | 0.640 |
| **POT** | **2.39** | **0.54** | **4.23** | **0.011** |
| **Control lens** | **2.36** | **0.14** | **4.58** | **0.037** |
| **Transmission** | **0.04** | **0.02** | **0.06** | **0.001** |
| **Contrast x migraine** | -0.90 | -2.18 | 0.38 | 0.168 |
| **Contrast x POT** | **-1.09** | **-1.81** | **-0.37** | **0.003** |
| **Contrast x control lens** | **-0.98** | **-1.70** | **-0.26** | **0.008** |
| Migraine x POT | -0.66 | -2.58 | 1.27 | 0.502 |
| Migraine x control lens | -0.12 | -2.14 | 1.90 | 0.905 |
| **Contrast x migraine x POT** | **1.19** | **0.16** | **2.23** | **0.024** |
| Contrast x migraine x control lens | 1.02 | -0.02 | 2.06 | 0.054 |

Background activity was also analysed using linear mixed effects model, which explained 92% of the variance. There was a main effect of MA group only, increased background activity in the MA compared to control group. The full table of results can be seen in Supplementary Table 4.

*Supplementary Table 4: Results for the background activity analysis using linear mixed effects model. Migraine is compared to the control group as a reference. The no lens condition is used as the reference for comparison for the POT and control lenses.*

|  | Coefficient estimate | Lower CI | Upper CI | p-value |
| --- | --- | --- | --- | --- |
| Contrast | -15.15 | -43.13 | 12.83 | 0.288 |
| **Migraine** | **498.70** | **160.33** | **837.07** | **0.004** |
| POT | 33.66 | -246.96 | 314.29 | 0.814 |
| Control lens | 103.85 | -259.31 | 467.01 | 0.575 |
| Transmission | 0.88 | -3.41 | 5.16 | 0.688 |

4.1) Experiment 1: Daylight Locus Choice of Colour

Based on previous literature it was expected that participants with MA would choose more saturated colours compared to the control group, however, this was not the case in the current experiment. This can be seen in Supplementary Figure 8. The curved line indicates the daylight locus. The further from the daylight locus, the more saturated the colours. We predicted that the MA group would choose colours further from the line compared to the control group, but this does not appear to be the case.


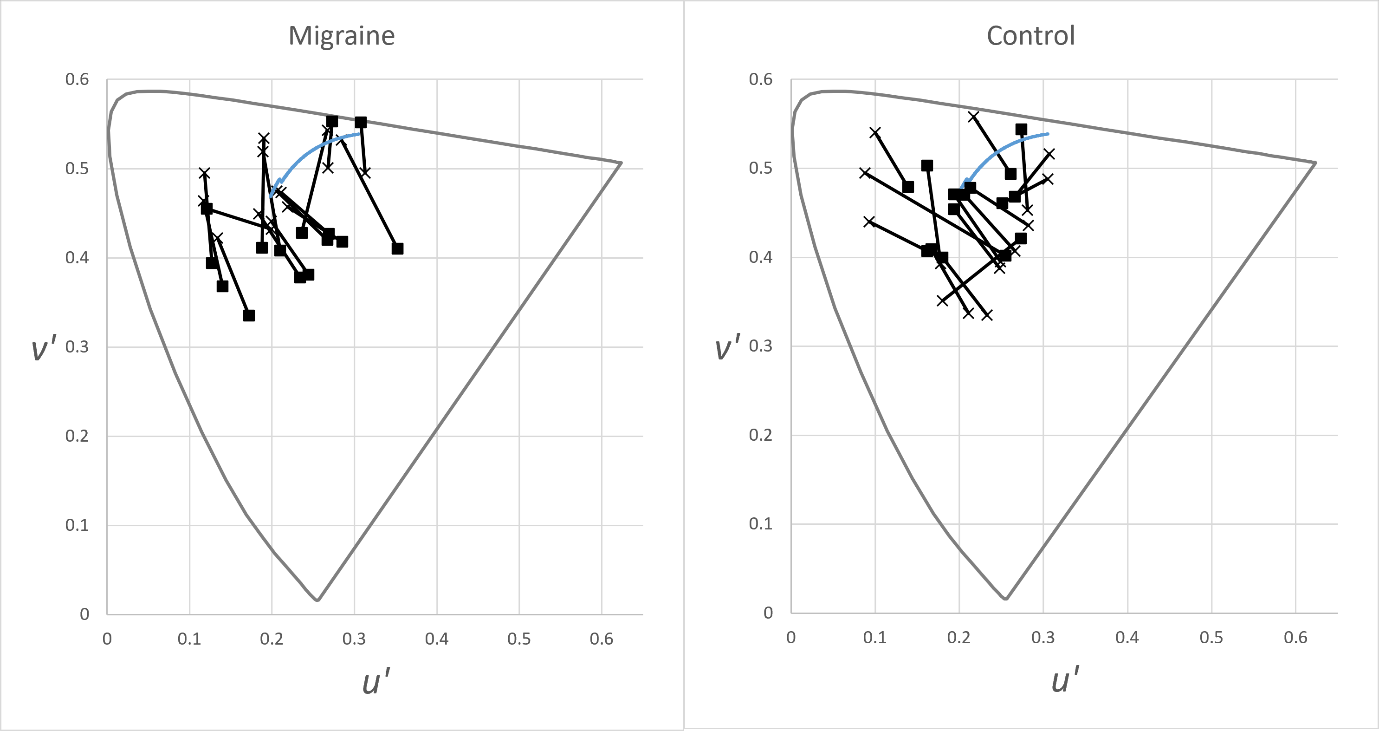


*Supplementary Figure 8: Choice of precision ophthalmic tints and corresponding control lenses for (left) MA and (right) control participants in CIE colour space (CIE 1976 Uniform Chromaticity Scale diagram) for Experiment 1. Squares indicate chosen lens and crosses indicate control lens. The curved line indicates the daylight locus (2000K-6500K).*

*Supplementary Table 5: Chromaticities in CIE u’v’ colour space, as well as estimated transmission, chosen by each of the participant for the Precision Ophthalmic Tint (POT) and control lens. MA = migraine aura.*

|  | POT | | | Control lens | | |
| --- | --- | --- | --- | --- | --- | --- |
| Observer type | u' | v' | transmission | u' | v' | transmission |
| MA | 0.199 | 0.432 | 30% | 0.121 | 0.455 | 13% |
| MA | 0.219 | 0.457 | 61% | 0.285 | 0.418 | 19% |
| MA | 0.199 | 0.441 | 38% | 0.244 | 0.381 | 16% |
| MA | 0.118 | 0.495 | 20% | 0.127 | 0.394 | 13% |
| MA | 0.190 | 0.534 | 50% | 0.188 | 0.411 | 27% |
| MA | 0.267 | 0.543 | 45% | 0.236 | 0.428 | 34% |
| MA | 0.313 | 0.495 | 23% | 0.308 | 0.552 | 31% |
| MA | 0.211 | 0.473 | 87% | 0.269 | 0.427 | 19% |
| MA | 0.268 | 0.501 | 32% | 0.273 | 0.553 | 36% |
| MA | 0.184 | 0.449 | 40% | 0.234 | 0.378 | 16% |
| MA | 0.189 | 0.519 | 50% | 0.210 | 0.408 | 22% |
| MA | 0.206 | 0.475 | 84% | 0.267 | 0.420 | 18% |
| MA | 0.134 | 0.422 | 16% | 0.172 | 0.335 | 16% |
| MA | 0.117 | 0.464 | 16% | 0.140 | 0.368 | 13% |
| MA | 0.284 | 0.532 | 35% | 0.352 | 0.410 | 13% |
| Control | 0.273 | 0.421 | 15% | 0.180 | 0.351 | 11% |
| Control | 0.248 | 0.466 | 34% | 0.305 | 0.488 | 26% |
| Control | 0.162 | 0.503 | 35% | 0.178 | 0.393 | 20% |
| Control | 0.194 | 0.471 | 55% | 0.249 | 0.395 | 23% |
| Control | 0.120 | 0.522 | 21% | 0.088 | 0.495 | 9% |
| Control | 0.139 | 0.479 | 22% | 0.100 | 0.540 | 10% |
| Control | 0.217 | 0.483 | 43% | 0.281 | 0.453 | 22% |
| Control | 0.194 | 0.454 | 51% | 0.248 | 0.388 | 22% |
| Control | 0.206 | 0.470 | 84% | 0.266 | 0.407 | 22% |
| Control | 0.215 | 0.478 | 89% | 0.282 | 0.436 | 26% |
| Control | 0.266 | 0.468 | 41% | 0.307 | 0.516 | 34% |
| Control | 0.261 | 0.494 | 38% | 0.217 | 0.558 | 43% |
| Control | 0.162 | 0.407 | 21% | 0.093 | 0.440 | 7% |
| Control | 0.167 | 0.409 | 20% | 0.211 | 0.337 | 12% |
| Control | 0.180 | 0.400 | 21% | 0.233 | 0.335 | 10% |

4.2) Experiment 1: Daylight Locus Analysis Assumptions

As the effect of lenses may well be influenced by the saturation of the lens, a separate analysis was conducted, this time analysing the SSVEP for the group that chose the more saturated colours compared to the group that chose chromaticities closer to the daylight locus. A median split was used to identify the two groups. Groups were defined as those who chose more saturated colours as the more extreme group, and those who chose colours closer to the Daylight locus. Supplementary Table 5 shows the chromaticities chosen by the participants. Supplementary Table 6 shows the model selection process for the linear mixed effect model, the best fitting model with the fewest parameters was the interaction model, which significantly outperformed the main effects only model. A significant interaction model emerged, accounting for 70% of the variance, including contrast, lens and group and their interactions as fixed effects, transmission as a covariate, and observer as a random effect (intercept). Supplementary Figure 9 shows the residuals for the model.

*Supplementary Table 6: Model build table, showing comparison to simpler models. RE = random effects, FE = fixed effects, LRT = simulated likelihood ratio test, AIC = Akaike Information Criterion, BIC = Bayes Information Criterion, LL = log-likelihood, df = degrees of freedom. All models are statistically significantly different from the null model.*

| Model specification | Model name | Nested /simpler model | Fixed effects added |  | Model Fit | | | | LRT Test | | |
| --- | --- | --- | --- | --- | --- | --- | --- | --- | --- | --- | --- |
|  | | | |  | AIC | BIC | LL | df | df | Χ^2^ | p |
| RE only | Null | - | - |  |  |  |  | 4 |  |  |  |
| **FE main effects** | **Main effects** | **Null** | **Contrast + lens + group** |  | **4171.5** | **4190.3** | **-2081.8** | **8** | **4** | **294.7** | **<0.001** |
| Interaction | Interactions | Main effects | Contrast * lens * group |  | 3884.8 | 3922.3 | -1934.4 | 15 | 7 | 8.13 | 0.32 |
| **FE main effects (random slope contrast)** | **Main effects** | **Main effects (intercept only)** | **Contrast + lens + group** |  | **3764.6** | **3811.6** | **-1872.3** | **10** | **2** | **124.2** | **<0.001** |
| **FE main effects (random slope contrast and lens)** | **Main effects** | **Main effects (intercept only)** | **Contrast + lens + group** |  | **3702.4** | **3777.5** | **-1835.2** | **16** | **8** | **198.4** | **<0.001** |


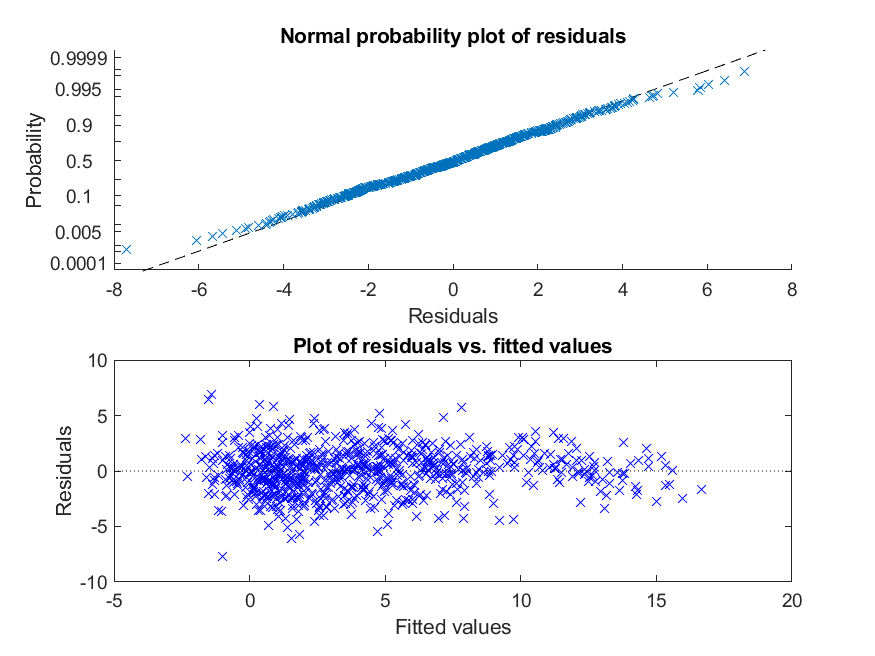


*Supplementary Figure 9: Top row shows the probability of normal residuals from the linear mixed effects model for the colours chosen (extreme or those close to the daylight locus) (normal distribution represented by the dotted line), bottom shows residuals against fitted values from the linear mixed effects model of the colours chosen (extreme or those close to the daylight locus).*

4.3) Experiment 1: Daylight Locus Results

Supplementary Figure 10 shows the spectra (top row) and the SSVEP against increasing contrast for the groups who chose the more extreme colours compared to those closer to the daylight locus.


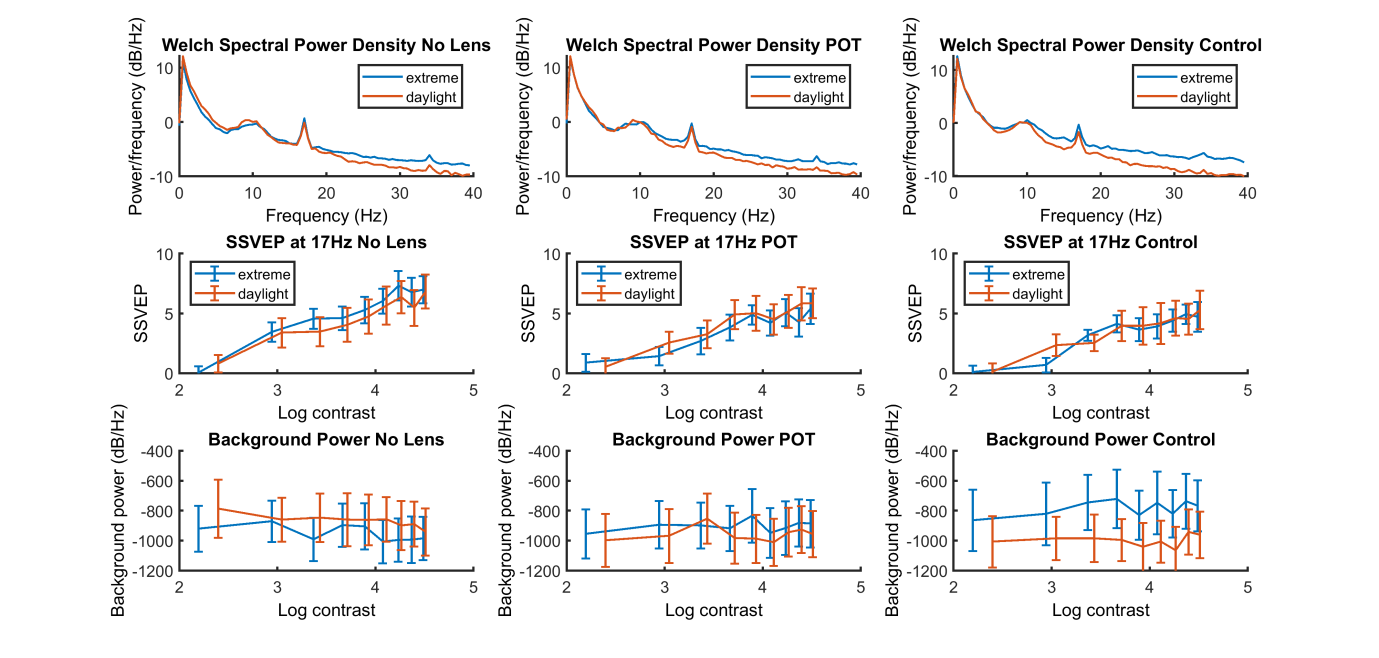


*Supplementary Figure 10: Top row shows spectral power density functions for both those who chose more saturated colours (extreme) and those who chose colours closer to the daylight locus for (left) no lens, (centre) POT and (right) control lens conditions. Middle row shows SSVEP against log contrast for those who chose more saturated colours (extreme) and those who chose colours closer to the daylight locus for (left) no lens, (centre) POT and (right) control lens conditions. Bottom row shows background noise (average of surrounding frequencies) against log contrast for those who chose more saturated colours (extreme) and those who chose colours closer to the daylight locus for (left) no lens, (centre) POT and (right) control lens conditions. Error bars indicate ± 1SE of the mean.*

A significant interaction model emerged, accounting for 80% of the variance including random intercept and slopes for contrast and lens. There was a main effect of contrast and transmission only. The complete table of results can be seen in Supplementary Table 7.

*Supplementary Table 7: Results for SSVEP of those choosing more saturated lenses, compared to those choosing lenses nearer to the daylight locus. The group choosing more extreme colours were compared to the group choosing colours closer to the daylight locus as the reference. The no lens condition is used as the reference for comparison for the POT and control lenses.*

|  | Coefficient estimate | Lower CI | Upper CI | p-value |
| --- | --- | --- | --- | --- |
| **Contrast** | **2.41** | **1.86** | **2.96** | **<0.001** |
| Extreme | -0.38 | -1.36 | 0.59 | 0.441 |
| POT | 0.69 | -0.70 | 2.08 | 0.332 |
| Control lens | 0.78 | -1.05 | 2.61 | 0.401 |
| **Transmission** | **0.03** | **0.01** | **0.05** | **0.010** |

The background activity was also analysed, a significant random interaction and random slope model emerged accounting for 91% of the variance. There was an interaction between contrast and control lens, and a significant interaction between the group who chose the most saturated colours (extreme) and control lens. The full set of results can be seen in Supplementary Table 8.

*Supplementary Table 8: Results for background activity of those choosing more saturated lenses, compared to those choosing lenses nearer to the daylight locus. The group choosing more extreme colours were compared to the group choosing colours closer to the daylight locus as the reference. The no lens condition is used as the reference for comparison for the POT and control lenses.*

|  | Coefficient estimate | Lower CI | Upper CI | p-value |
| --- | --- | --- | --- | --- |
| Contrast | -50.50 | -107.26 | 6.26 | 0.081 |
| Extreme | -143.03 | -588.50 | 302.45 | 0.529 |
| POT | -214.44 | -510.30 | 81.43 | 0.155 |
| Control lens | -229.02 | -631.81 | 173.77 | 0.265 |
| Transmission | 0.02 | -4.52 | 4.55 | 0.995 |
| Contrast x extreme | 7.14 | -73.13 | 87.42 | 0.861 |
| Contrast x POT | 56.77 | -10.46 | 124.01 | 0.098 |
| **Contrast x control lens** | **79.15** | **11.91** | **146.39** | **0.021** |
| Extreme x POT | 173.49 | -82.91 | 429.88 | 0.185 |
| **Extreme x control lens** | **282.79** | **26.15** | **539.43** | **0.031** |
| Contrast x extreme x POT | 6.56 | -88.53 | 101.65 | 0.892 |
| Contrast x extreme x control lens | 1.87 | -93.22 | 96.96 | 0.969 |

**5) Experiment 2**

5.1) Experiment 2: SSVEP at 5Hz

A histogram of the distribution of the SSVEP responses at 5Hz can be seen in Supplementary Figure 11. The skewness is 0.44. A significant interaction model including main effects of contrast, lens and group, and a random effect of observer (intercept and random slope of contrast and lens) gave a better fit (compared to main effects only model, based on log ratio test). Supplementary Figure 12 shows the distribution of residuals for the chosen linear mixed effects model.


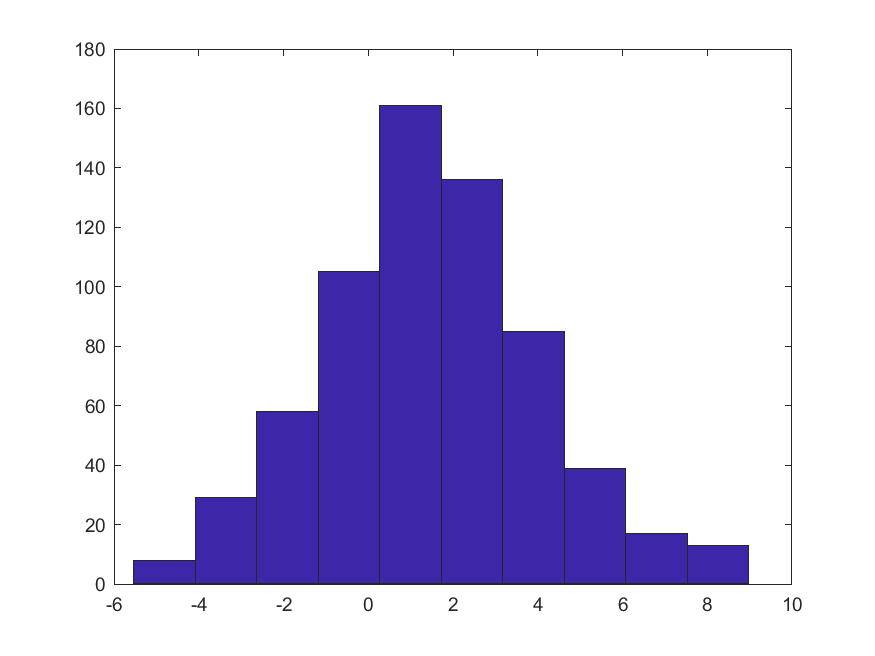


*Supplementary Figure 11: Histogram of the SSVEP activity at 5Hz.*

Model building process: SSVEP ~ contrast * lens * group + (1|observer)

*Supplementary Table 9: Model build table, showing comparison to simpler models. RE = random effects, FE = fixed effects, LRT = simulated likelihood ratio test, AIC = Akaike Information Criterion, BIC = Bayes Information Criterion, LL = log-likelihood, df = degrees of freedom. All models are statistically significantly different from the null model.*

| Model specification | Model name | Nested /simpler model | Fixed effects added |  | Model Fit | | | | LRT Test | | |
| --- | --- | --- | --- | --- | --- | --- | --- | --- | --- | --- | --- |
|  | | | |  | AIC | BIC | LL | df | df | Χ^2^ | p |
| RE only | Null | - | - |  | 3336.5 | 3354.4 | -1664.3 | 4 |  |  |  |
| **FE main effects** | **Main effects** | **Null** | **Contrast + lens + group** |  | 3109.80 | 3145.6 | -1546.9 | **8** | **4** | **234.7** | **<0.001** |
| Interaction | Interaction | Main effects | Contrast * lens * group |  | 3120.8 | 3187.8 | -1545.4 | 15 | 7 | 3.1 | 0.88 |
| **FE main effects (random slope contrast)** |  | **Main effects (interaction only)** | **Contrast + lens + group** |  | **3052.3** | **3096.9** | **-1516.1** | **10** | **2** | **61.6** | **<0.001** |
| **FE main effects (random slope contrast and lens)** |  | **Main effects (interaction only)** | **Contrast + lens + group** |  | **3001.1** | **3072.6** | **-1484.5** | **16** | **8** | **124.8** | **<0.001** |


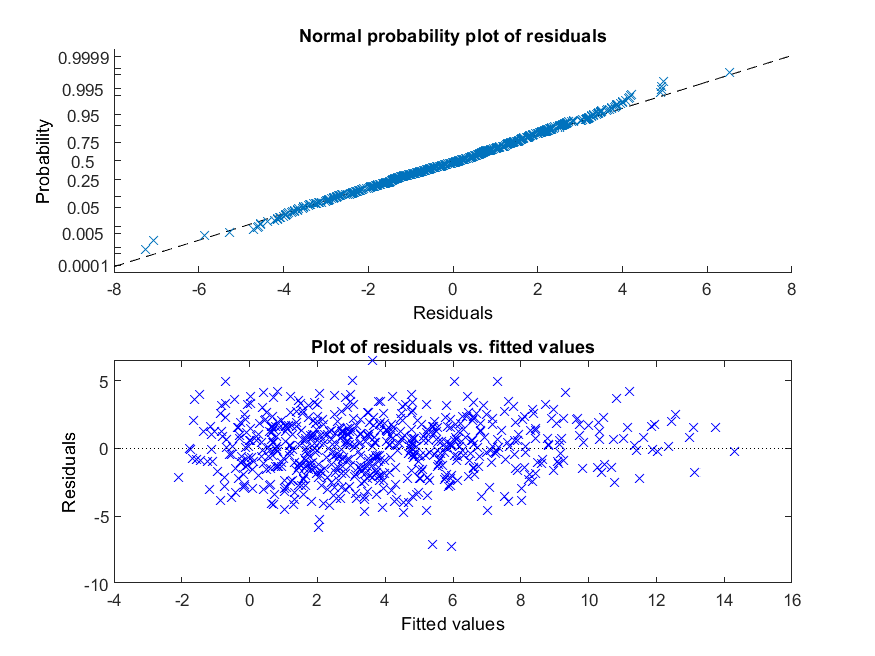


*Supplementary Figure 12: Top shows the probability of normal residuals from the linear mixed effects model for the SSVEP (normal distribution represented by the dotted line), bottom shows residuals against fitted values from the linear mixed effects model of the SSVEP.*

5.2) Experiment 2: 5Hz Background Activity

Supplementary Figure 13 shows the distribution of background activity in the 5Hz condition for Experiment 2. The best fitting model with the fewest parameters was the main effects model, in effects of contrast, group and lens, and a random effect of observer (including random intercept and slope for contrast and lens), see Supplementary Table 10. There was no statistically significant improvement on the model from including the interaction terms. Distribution of residuals can be seen in Supplementary Figure 14.


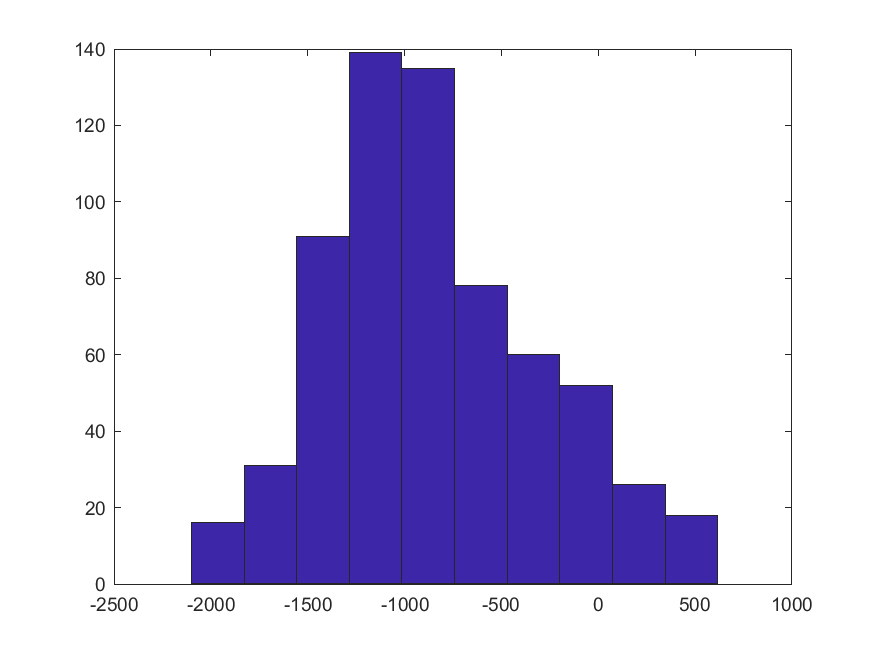


*Supplementary Figure 13: Histogram of the background activity, total over 6 to 30Hz, omitting 5Hz as the stimulation frequency.*

*Supplementary Table 10: Model build table, showing comparison to simpler models. RE = random effects, FE = fixed effects, LRT = simulated likelihood ratio test, AIC = Akaike Information Criterion, BIC = Bayes Information Criterion, LL = log-likelihood, df = degrees of freedom. All models are statistically significantly different from the null model.*

| Model specification | Model name | Nested /simpler model | Fixed effects added |  | Model Fit | | | | LRT Test | | |
| --- | --- | --- | --- | --- | --- | --- | --- | --- | --- | --- | --- |
|  | | | |  | AIC | BIC | LL | df | df | Χ^2^ | p |
| RE only | Null | - | - |  | 9191.4 | 9209.3 | -4591.7 | 4 |  |  |  |
| **FE main effects** | **Main effects** | **Null** | **Contrast + lens + group** |  | **9122.5** | **9158.3** | **-4553.3** | **8** | **4** | **76.9** | **<0.001** |
| Interactions | Interactions | Main effects | Contrast * lens * group |  | 9129.0 | 9196.0 | -4549.5 | 15 | 7 | 7.6 | 0.37 |
| **FE main effects (random slope contrast)** | **Main effects** | **Main effects (interaction only)** | **Contrast + lens + group** |  | **9091.1** | **9135.8** | **-4535.5** | **10** | **2** | **35.5** | **<0.001** |
| **FE main effects (random slope contrast + lens)** | **Main effects** | **Main effects (interaction only)** | **Contrast + lens + group** |  | **8866.1** | **8937.7** | **-4417.1** | **16** | **8** | **272.4** | **<0.001** |


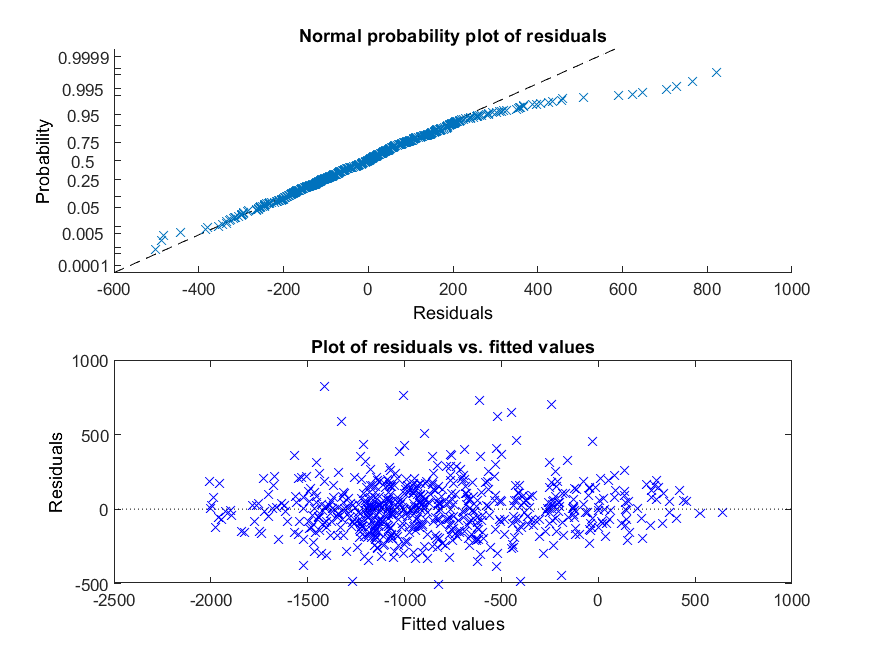


*Supplementary Figure 14: Top shows the probability of normal residuals from the linear mixed effects model for the background activity (normal distribution represented by the dotted line), bottom shows residuals against fitted values from the linear mixed effects model of the background activity.*

5.3) Experiment 2: 5Hz Distance from Daylight Locus

As in the Experiment 1, the MA group did not choose more saturated colours compared to the control group. Therefore, a median split was used to identify the two groups: groups were defined as those who chose more saturated colours as the more extreme group, and those who chose colours closer to the Daylight locus. Supplementary Figure 15 shows the colour choice in CIE colour space. Based on previous literature, the MA group choices are no further from the daylight locus (blue line) compared to the control group colour choices. The chromaticities of the choice of colours can be seen in Supplementary Table 11.


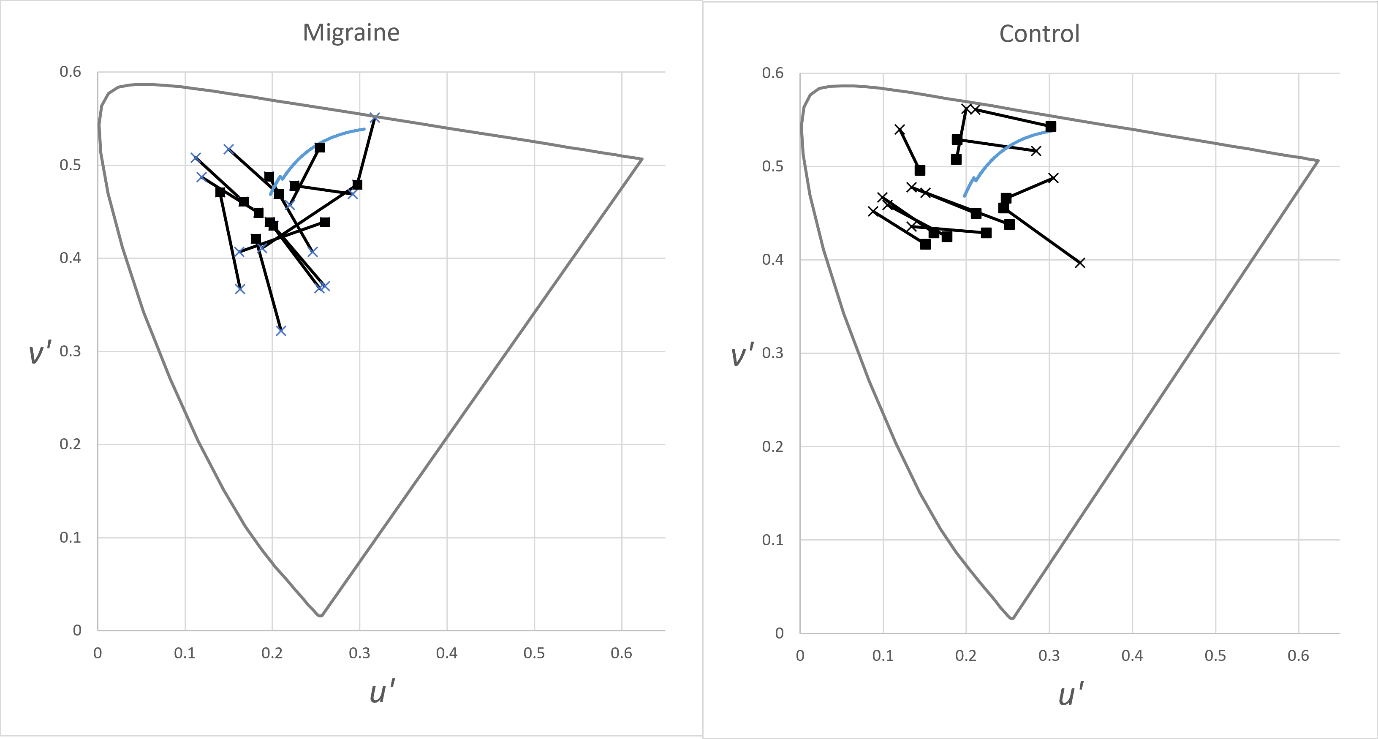


*Supplementary Figure 15: Choice of precision ophthalmic tints and corresponding control lenses for (left) MA and (right) control participants in CIE colour space (CIE 1976 Uniform Chromaticity Scale diagram) for Experiment 2. Squares indicate chosen lens and crosses indicate control lens. The curved line indicates the daylight locus (2000K-6500K).*

*Supplementary Table 11: Chromaticities in CIE u’v’ colour space, as well as transmission, chosen by each of the participant for the Precision Ophthalmic Tint (POT) and control lens. MA = migraine aura.*

|  | POT | | | Control lens | | |
| --- | --- | --- | --- | --- | --- | --- |
| Observer type | u' | v' | transmission | u' | v' | transmission |
| MA | 0.297 | 0.479 | 27% | 0.188 | 0.411 | 27% |
| MA | 0.297 | 0.479 | 27% | 0.317 | 0.551 | 29% |
| MA | 0.254 | 0.519 | 65% | 0.220 | 0.457 | 53% |
| MA | 0.140 | 0.471 | 22% | 0.163 | 0.367 | 22% |
| MA | 0.260 | 0.439 | 28% | 0.162 | 0.407 | 21% |
| MA | 0.207 | 0.469 | 73% | 0.150 | 0.517 | 26% |
| MA | 0.196 | 0.488 | 57% | 0.246 | 0.407 | 29% |
| MA | 0.167 | 0.461 | 36% | 0.112 | 0.508 | 15% |
| MA | 0.225 | 0.478 | 63% | 0.292 | 0.469 | 27% |
| MA | 0.184 | 0.449 | 40% | 0.119 | 0.487 | 14% |
| MA | 0.201 | 0.435 | 31% | 0.260 | 0.370 | 17% |
| MA | 0.197 | 0.439 | 36% | 0.254 | 0.368 | 20% |
| MA | 0.181 | 0.421 | 31% | 0.210 | 0.322 | 8% |
| C | 0.189 | 0.529 | 56% | 0.284 | 0.517 | 41% |
| C | 0.177 | 0.425 | 36% | 0.105 | 0.459 | 8% |
| C | 0.252 | 0.438 | 33% | 0.151 | 0.472 | 20% |
| C | 0.224 | 0.429 | 36% | 0.134 | 0.436 | 17% |
| C | 0.188 | 0.508 | 56% | 0.200 | 0.562 | 24% |
| C | 0.302 | 0.543 | 39% | 0.211 | 0.561 | 36% |
| C | 0.161 | 0.429 | 29% | 0.099 | 0.467 | 10% |
| C | 0.248 | 0.466 | 37% | 0.305 | 0.488 | 26% |
| C | 0.151 | 0.417 | 19% | 0.088 | 0.452 | 6% |
| C | 0.144 | 0.496 | 30% | 0.120 | 0.540 | 16% |
| C | 0.212 | 0.450 | 48% | 0.134 | 0.478 | 25% |
| C | 0.245 | 0.456 | 38% | 0.337 | 0.397 | 7% |

Model selection can be seen in Supplementary Table 12. Based on log ratio test, the best fitting model was the main effect only model including contrast, group and lens as main effects, and observer as a random effect. There was no improvement from including the interactions. Supplementary Figure 16 shows the distribution of residuals.

*Supplementary Table 12: Model build table, showing comparison to simpler models. RE = random effects, FE = fixed effects, LRT = simulated likelihood ratio test, AIC = Akaike Information Criterion, BIC = Bayes Information Criterion, LL = log-likelihood, df = degrees of freedom. All models are statistically significantly different from the null model.*

| Model specification | Model name | Nested /simpler model | Fixed effects added |  | Model Fit | | | | | LRT Test | | | |
| --- | --- | --- | --- | --- | --- | --- | --- | --- | --- | --- | --- | --- | --- |
|  | | | |  | AIC | BIC | LL | df | df | | Χ^2^ | p |  |
| RE only | Null | - | - |  | 3336.5 | 3354.4 | -1664.3 | 4 |  | |  |  |  |
| **FE main effects** | **Main effects** | **Null** | **Contrast + lens + group** |  | **3108.4** | **3144.1** | **-1546.2** | **8** | **4** | | **236.2** | **<0.001** |  |
| Interactions | Interactions | Main effects | Contrast * lens * group |  | 3116.5 | 3183.5 | -1543.3 | 15 | 7 | | 5.9 | 0.555 |  |
| **FE main effects (random slope contrast)** | **Main effects** | **Main effects (intercept only)** | **Contrast + lens + group** |  | **3051.2** | **3095.9** | **-1515.6** | **10** | **2** | | **61.2** | **<0.001** |  |
| **FE main effects (random slope contrast and lens)** | **Main effects** | **Main effects (intercept only)** | **Contrast + lens + group** |  | **3000.2** | **3071.7** | **-1484.1** | **16** | **8** | | **124.2** | **<0.001** |  |


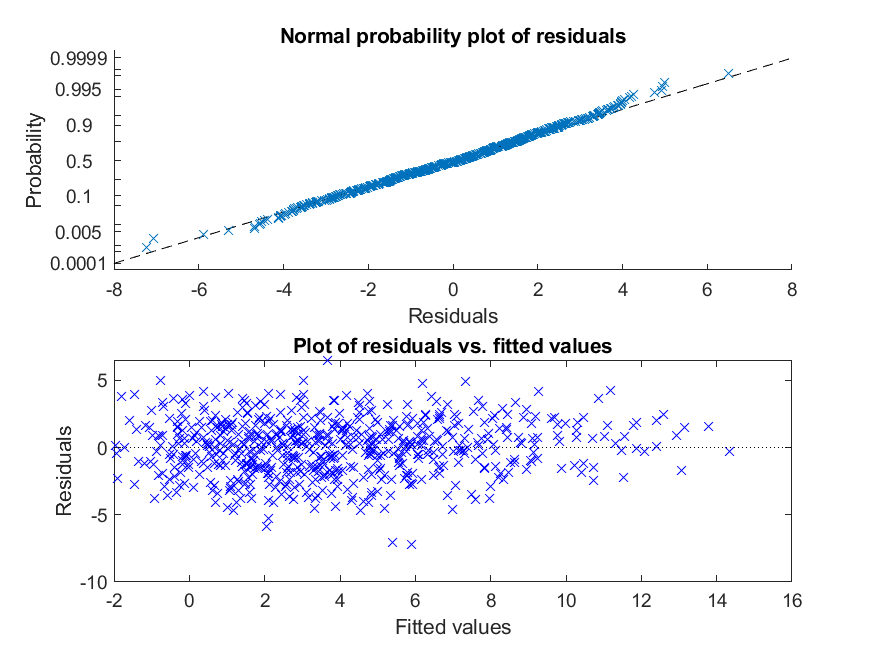


*Supplementary Figure 16: Top shows the probability of normal residuals from the linear mixed effects model for the SSVEP (normal distribution represented by the dotted line), bottom shows residuals against fitted values from the linear mixed effects model of the SSVEP.*

Supplementary Figure 17 shows the spectra (top row) and the SSVEP against increasing contrast for the groups who chose the more extreme colours compared to those with choice closer to the daylight locus.


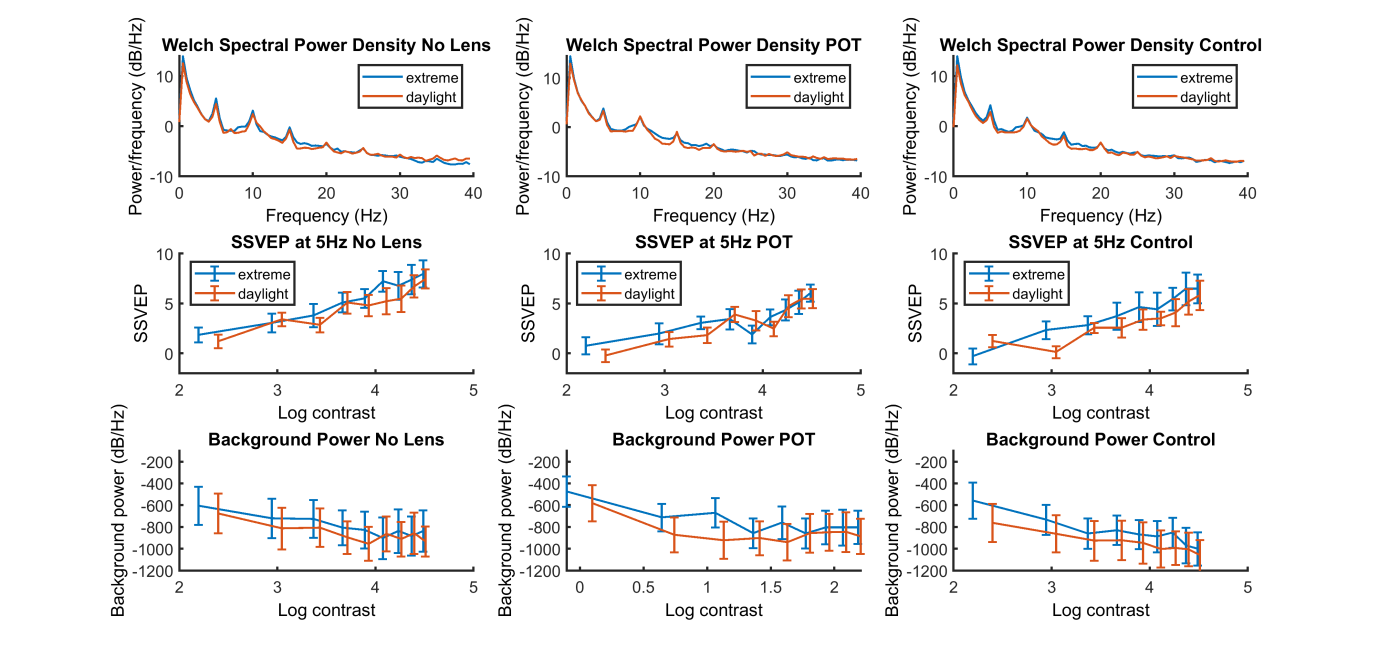


*Supplementary Figure 17: Top row shows spectral power density functions for both those who chose more saturated colours (extreme) and those who chose colours closer to the daylight locus for (left) no lens, (centre) POT and (right) control lens conditions. Middle row shows SSVEP against log contrast for those who chose more saturated colours (extreme) and those who chose colours closer to the daylight locus for (left) no lens, (centre) POT and (right) control lens conditions. Bottom row shows background noise (average of surrounding frequencies) against log contrast for those who chose more saturated colours (extreme) and those who chose colours closer to the daylight locus for (left) no lens, (centre) POT and (right) control lens conditions. Error bars indicate ± 1SE of the mean.*

A statistically significant model emerged including main effects only, as there was no statistically significant improvement in the model when interaction terms were included. The model accounted for 74% of the variance. The full table of results of the linear mixed effect model can be seen in Supplementary Table 13. There was a main effect of contrast only, results showed that increasing stimulus contrast resulted in increasing SSVEP.

*Supplementary Table 13: Results of the linear mixed effect model for SSVEP for 5Hz stimulation frequency. Those who chose extreme values (saturated colours) are compared to those who chose less saturated colours, closer to the daylight locus as a reference. The no lens condition is used as the reference for comparison for the POT and control lenses.*

|  | Coefficient estimate | Lower CI | Upper CI | p-value |
| --- | --- | --- | --- | --- |
| **Contrast** | **2.48** | **1.92** | **3.05** | **<0.001** |
| Extreme | 0.81 | -0.32 | 1.94 | 0.159 |
| POT | -0.25 | -2.73 | 2.23 | 0.842 |
| Control lens | 0.46 | -2.60 | 3.52 | 0.769 |
| Transmission | 0.03 | -0.01 | 0.06 | 0.200 |

A linear mixed effects model was also conducted to assess background activity, as in Experiment 1. A statistically significant model emerged for background activity, accounting for 90% of the variance. The best fitting model was a random slope model, included only main effects, based on log ratio test. There was a significant main effect of contrast only. The full table of results can be seen in Supplementary Table 14.

*Supplementary Table 14: Results of the linear mixed effect model for background activity for 5Hz stimulation frequency. Those who chose extreme values (saturated colours) are compared to those who chose less saturated colours, closer to the daylight locus as a reference. The no lens condition is used as the reference for comparison for the POT and control lenses.*

|  | Coefficient estimate | p-value | Lower CI | Upper CI |
| --- | --- | --- | --- | --- |
| **Contrast** | **-122.44** | **-177.91** | **-66.97** | **<0.001** |
| Extreme | 13.80 | -367.96 | 395.55 | 0.943 |
| POT | 66.62 | -312.67 | 445.91 | 0.730 |
| Control lens | -13.03 | -487.00 | 460.93 | 0.957 |
| Transmission | 0.63 | -5.28 | 6.55 | 0.834 |

5.4) Experiment 2: SSVEP at 17Hz

Supplementary Figure 18 shows the distribution of SSVEP responses at 17Hz. The skewness was -0.08. The best fitting model from the log ratio test was a random slope model and included main effects of contrast, group and lens and observer as a random effect, see Supplementary Table 15. Supplementary Figure 19 shows the distribution of residuals.


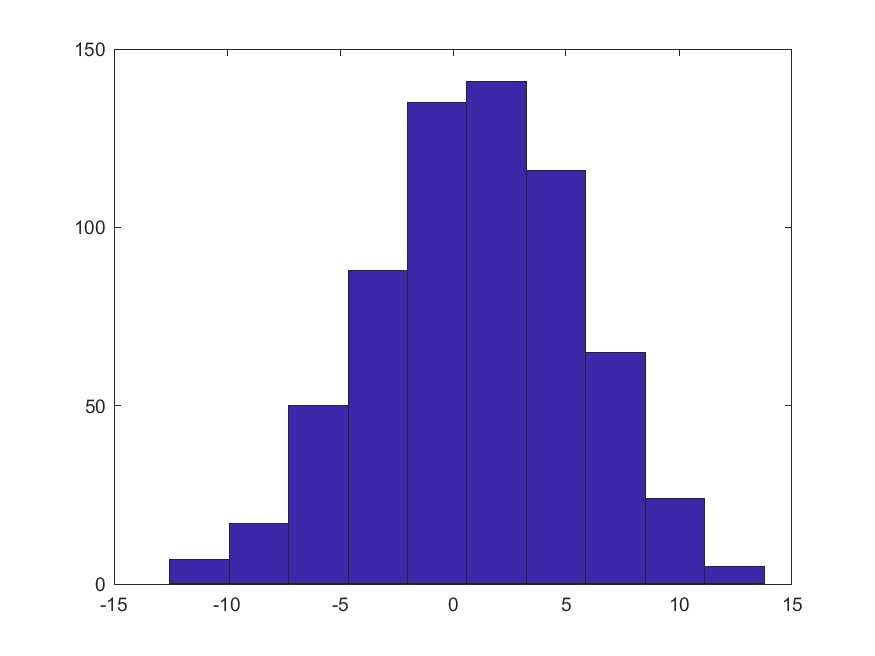


*Supplementary Figure 18: Histogram of the SSVEP response at 17Hz in Experiment 2.*

*Supplementary Table 15: Model build table, showing comparison to simpler models. RE = random effects, FE = fixed effects, LRT = simulated likelihood ratio test, AIC = Akaike Information Criterion, BIC = Bayes Information Criterion, LL = log-likelihood, df = degrees of freedom. All models are statistically significantly different from the null model.*

| Model specification | Model name | Nested /simpler model | Fixed effects added |  | Model Fit | | | | | LRT Test | | | |
| --- | --- | --- | --- | --- | --- | --- | --- | --- | --- | --- | --- | --- | --- |
|  | | | |  | AIC | BIC | LL | df | df | | Χ^2^ | p |  |
| RE only | Null | - | - |  | 3613.0 | 3630.9 | -1802.5 | 4 |  | |  |  |  |
| **FE main effects** | **Main effects** | **Null** | **Contrast + lens + group** |  | **3323.2** | **3359.0** | **-1653.6** | **8** | **4** | | **297.9** | **<0.001** |  |
| Interactions | Interactions | Main effects | Contrast * lens * group |  | 3324.5 | 3391.7 | -1647.3 | 15 | 7 | | 12.6 | 0.081 |  |
| **Interactions (random slope contrast)** | **Interactions** | **Interactions (intercept only)** | **Contrast * lens * group** |  | **3205.8** | **3281.8** | **-1585.9** | **17** | **2** | | **122.77** | **<0.001** |  |
| **Interactions (random slope contrast + lens)** | **Interactions** | **Interactions (intercept only)** | **Contrast * lens * group** |  | **3187.8** | **3290.7** | **-1570.9** | **23** | **8** | | **152.79** | **<0.001** |  |


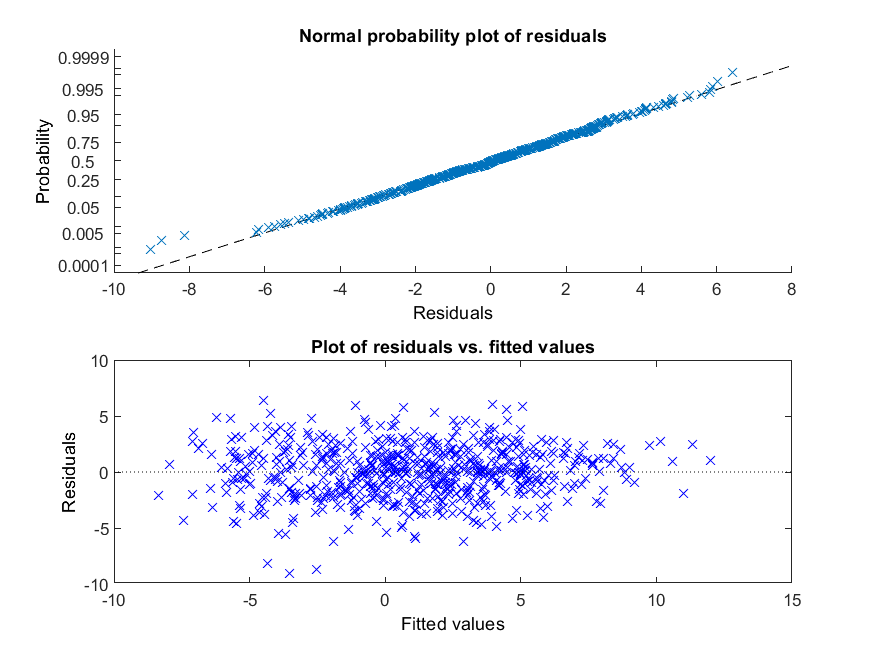


*Supplementary Figure 19: Top shows the probability of normal residuals from the linear mixed effects model for the SSVEP (normal distribution represented by the dotted line), bottom shows residuals against fitted values from the linear mixed effects model of the SSVEP.*

5.5) Experiment 2: Background Activity at 17Hz

Distribution can be seen in Supplementary Figure 20. Supplementary Table 16 shows the best fitting model (log ratio test) including main effects of contrast, group and lens as main effects and observer as a random effect (intercept). Distribution of residuals can be seen in Supplementary Figure 21.


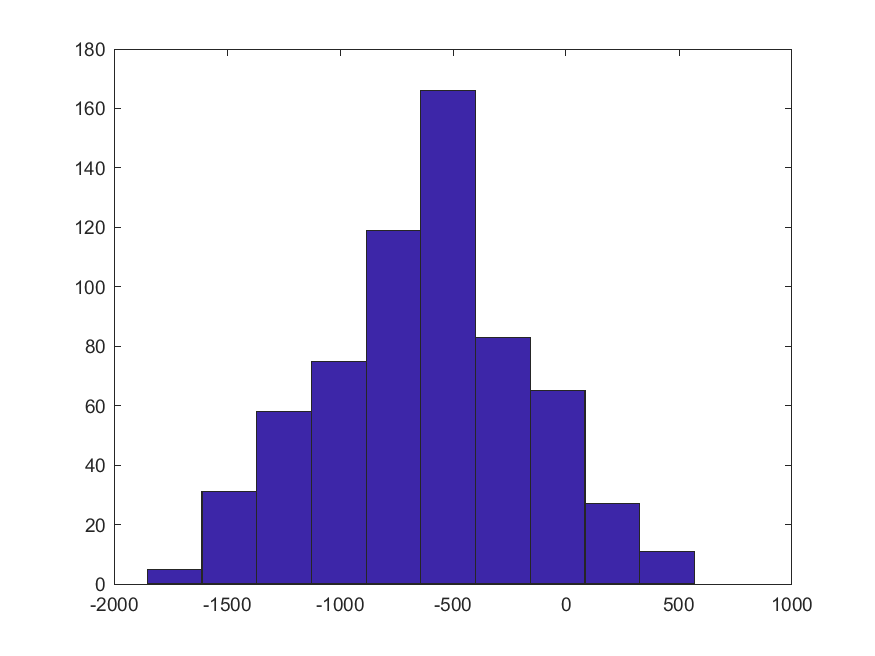


*Supplementary Figure 20: Histogram of the background activity, total over 6 to 30Hz, omitting 17Hz as the stimulation frequency.*

*Supplementary Table 16: Model build table, showing comparison to simpler models. RE = random effects, FE = fixed effects, LRT = simulated likelihood ratio test, AIC = Akaike Information Criterion, BIC = Bayes Information Criterion, LL = log-likelihood, df = degrees of freedom. All models are statistically significantly different from the null model.*

| Model specification | Model name | Nested /simpler model | Fixed effects added |  | Model Fit | | | | | LRT Test | | | |
| --- | --- | --- | --- | --- | --- | --- | --- | --- | --- | --- | --- | --- | --- |
|  | | | |  | AIC | BIC | LL | df | df | | Χ^2^ | p |  |
| RE only | Null | - | - |  | 9115.7 | 9133.5 | -4553.9 | 4 |  | |  |  |  |
| **FE main effects** | **Main effects** | **Null** | **Contrast + lens + group** |  | **8878.8** | **8914.5** | **-4431.4** | **8** | **4** | | **244.9** | **<0.001** |  |
| Interactions | Interactions | Main effects | Contrast * lens * group |  | 8883.9 | 8950.8 | -4426.9 | 15 | 7 | | 9.0 | 0.255 |  |
| **FE main effects (random slope contrast)** | **Main effects** | **Main effects (intercept only)** | **Contrast + lens + group** |  | **8860.9** | **8905.5** | **-4420.4** | **10** | **2** | | **21.95** | **<0.001** |  |
| **FE main effects (random slope contrast + lens)** | **Main effects** | **Main effects (intercept only)** | **Contrast + lens + group** |  | **8597.3** | **8668.7** | **-4282.7** | **16** | **8** | | **297.48** | **<0.001** |  |


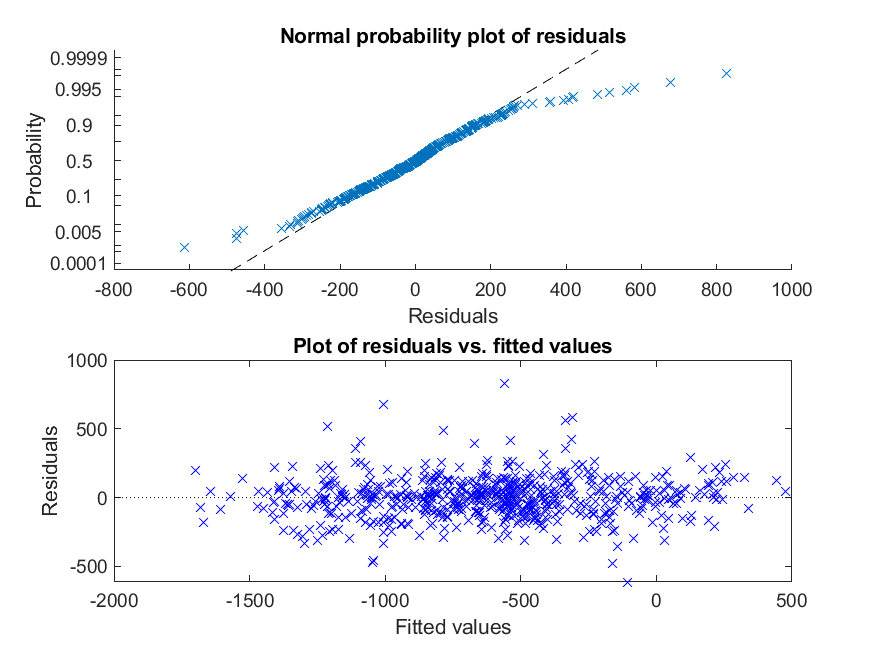


*Supplementary Figure 21: Top shows the probability of normal residuals from the linear mixed effects model for the background activity (normal distribution represented by the dotted line), bottom shows residuals against fitted values from the linear mixed effects model of the background activity.*

5.6) Experiment 2: Distance from Daylight Locus at 17Hz

As in Experiment 1, the MA group did not choose more saturated colours compared to the control group, which has already been discussed previously. Therefore, as in the 5Hz analysis, the SSVEP responses were analysed for the group who chose the more saturated colours compared to those closer to the daylight locus, based on median split. Supplementary Figure 22 shows the spectra (top row) and the SSVEP against increasing contrast for the groups who chose the more extreme colours compared to those closer to the daylight locus.


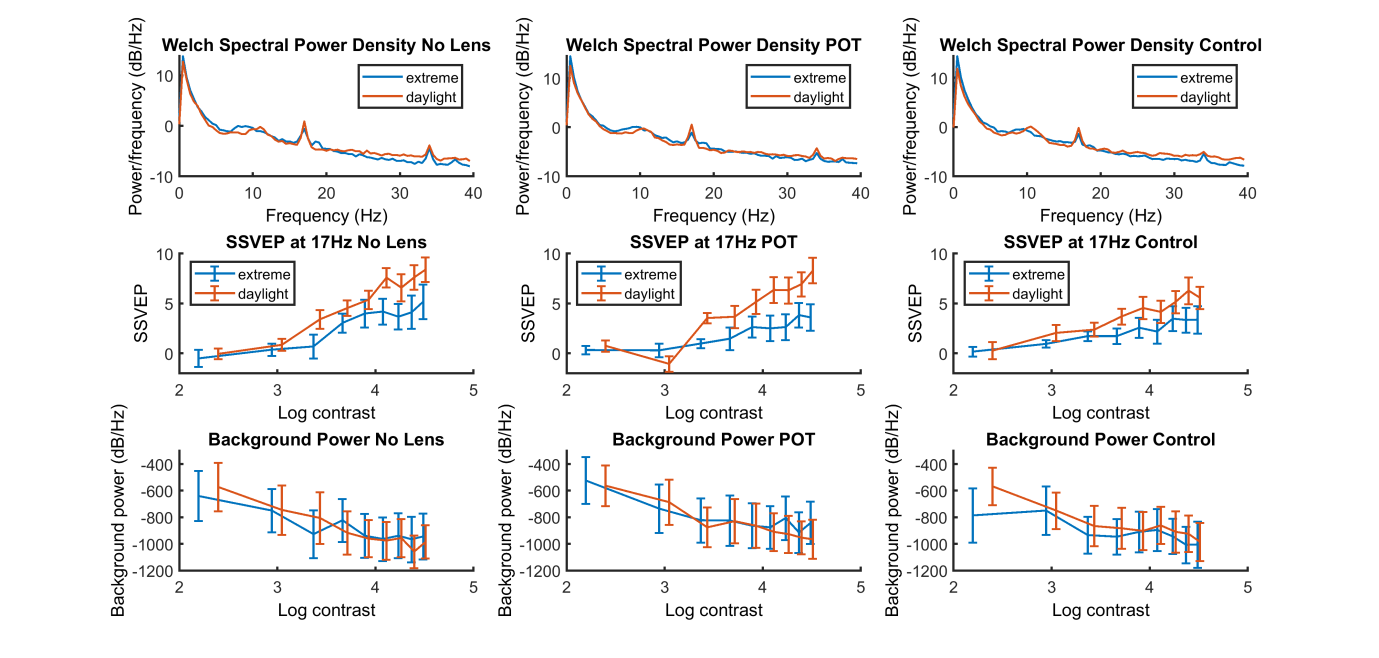


*Supplementary Figure 22: Top row shows spectral power density functions for both those who chose more saturated colours (extreme) and those who chose colours closer to the daylight locus for (left) no lens, (centre) POT and (right) control lens conditions. Middle row shows SSVEP against log contrast for those who chose more saturated colours (extreme) and those who chose colours closer to the daylight locus for (left) no lens, (centre) POT and (right) control lens conditions. Bottom row shows background noise (average of surrounding frequencies) against log contrast for those who chose more saturated colours (extreme) and those who chose colours closer to the daylight locus for (left) no lens, (centre) POT and (right) control lens conditions. Error bars indicate ± 1SE of the mean.*

Supplementary Table 17 shows the best fitting model including contrast, lens, group, and their interactions as fixed effects, transmission as a covariate, and observer as a random effect. The distribution of residuals of the chosen model can be seen in Supplementary Figure 23.

*Supplementary Table 17: Model build table, showing comparison to simpler models. RE = random effects, FE = fixed effects, LRT = simulated likelihood ratio test, AIC = Akaike Information Criterion, BIC = Bayes Information Criterion, LL = log-likelihood, df = degrees of freedom. All models are statistically significantly different from the null model.*

| Model specification | Model name | Nested /simpler model | Fixed effects added |  | Model Fit | | | | | LRT Test | | | |
| --- | --- | --- | --- | --- | --- | --- | --- | --- | --- | --- | --- | --- | --- |
|  | | | |  | AIC | BIC | LL | df | df | | Χ^2^ | p |  |
| RE only | Null | - | - |  | 3457.2 | 3475.1 | -1724.6 | 4 |  | |  |  |  |
| **FE main effects** | **Main effects** | **Null** | **Contrast + lens + group** |  | **3223.7** | **3259.4** | **-1603.8** | **8** | **4** | | **241.6** | **<0.001** |  |
| **Interactions** | **Interactions** | **Main effects** | **Contrast * lens * group** |  | **3195.6** | **3262.7** | **-1582.8** | **15** | **7** | | **42.0** | **<0.001** |  |
| **Interactions (random slope lens)** | **Interactions** | **Interactions (intercept only)** | **Contrast * lens * group** |  | **3013.1** | **3089.1** | **-1489.5** | **17** | **2** | | **186.52** | **<0.001** |  |
| **Interactions (random slope contrast and lens)** | **Interactions** | **Interactions (intercept only)** | **Contrast * lens * group** |  | **3005.4** | **3108.2** | **-1479.7** | **23** | **8** | | **206.26** | **<0.001** |  |


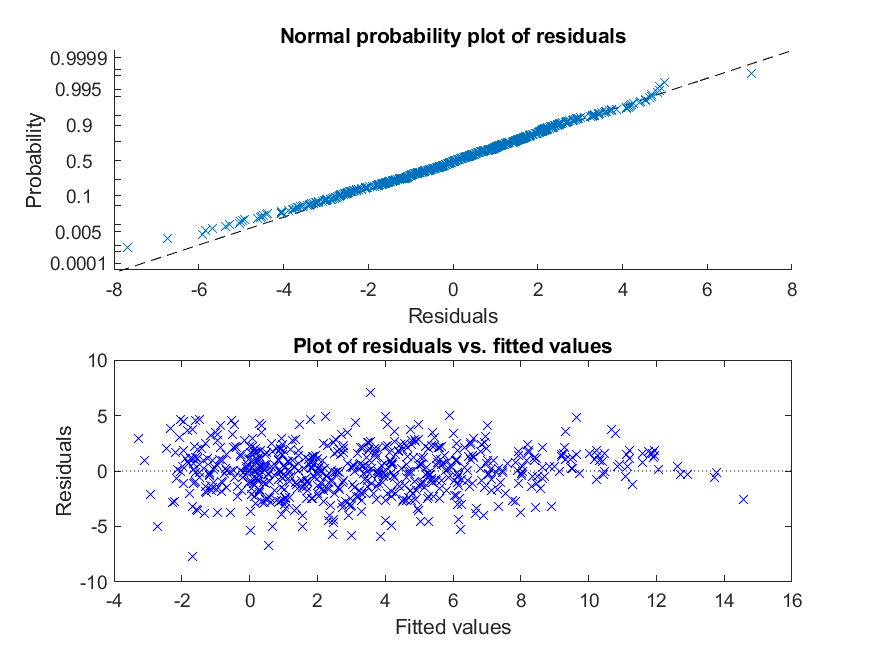


*Supplementary Figure 23: Top shows the probability of normal residuals from the linear mixed effects model for the SSVEP (normal distribution represented by the dotted line), bottom shows residuals against fitted values from the linear mixed effects model of the SSVEP.*

The model accounted for 74% of the variance. There was an increase in SSVEP with increasing stimulus contrast. There was a reduced effect of contrast for the people who chose more saturated colours compared to those who chose colours closer to the daylight locus. There was a reduced effect of contrast when using the control lens compared to no lens condition - the contrast response is flatter with the control lens only. The full set of results for the linear mixed effect model can be seen in Supplementary Table 18. The interaction effects can be seen more clearly in Supplementary Figure 24.

*Supplementary Table 18: Results of the linear mixed effect model for SSVEP for 17Hz stimulation frequency. Those who chose extreme values (saturated colours) are compared to those who chose less saturated colours, closer to the daylight locus as a reference. The no lens condition is used as the reference for comparison for the POT and control lenses.*

|  | Coefficient estimate | Lower CI | Upper CI | p-value |
| --- | --- | --- | --- | --- |
| **Contrast** | **4.08** | **2.79** | **5.38** | **<0.001** |
| Extreme | -0.06 | -1.60 | 1.48 | 0.938 |
| POT | 1.59 | -0.56 | 3.73 | 0.146 |
| Control lens | 3.59 | 0.77 | 6.40 | 0.013 |
| **Transmission** | **0.03** | **0.00** | **0.07** | **0.030** |
| Contrast x extreme | -1.53 | -3.37 | 0.31 | 0.103 |
| Contrast x POT | -0.21 | -1.06 | 0.64 | 0.626 |
| **Contrast x control lens** | **-1.49** | **-2.34** | **-0.64** | **0.001** |
| Extreme x POT | 1.36 | -0.73 | 3.46 | 0.201 |
| Extreme x control lens | 0.17 | -2.04 | 2.39 | 0.877 |
| Contrast x extreme x POT | -0.64 | -1.85 | 0.56 | 0.295 |
| Contrast x extreme x control lens | 0.47 | -0.74 | 1.67 | 0.448 |


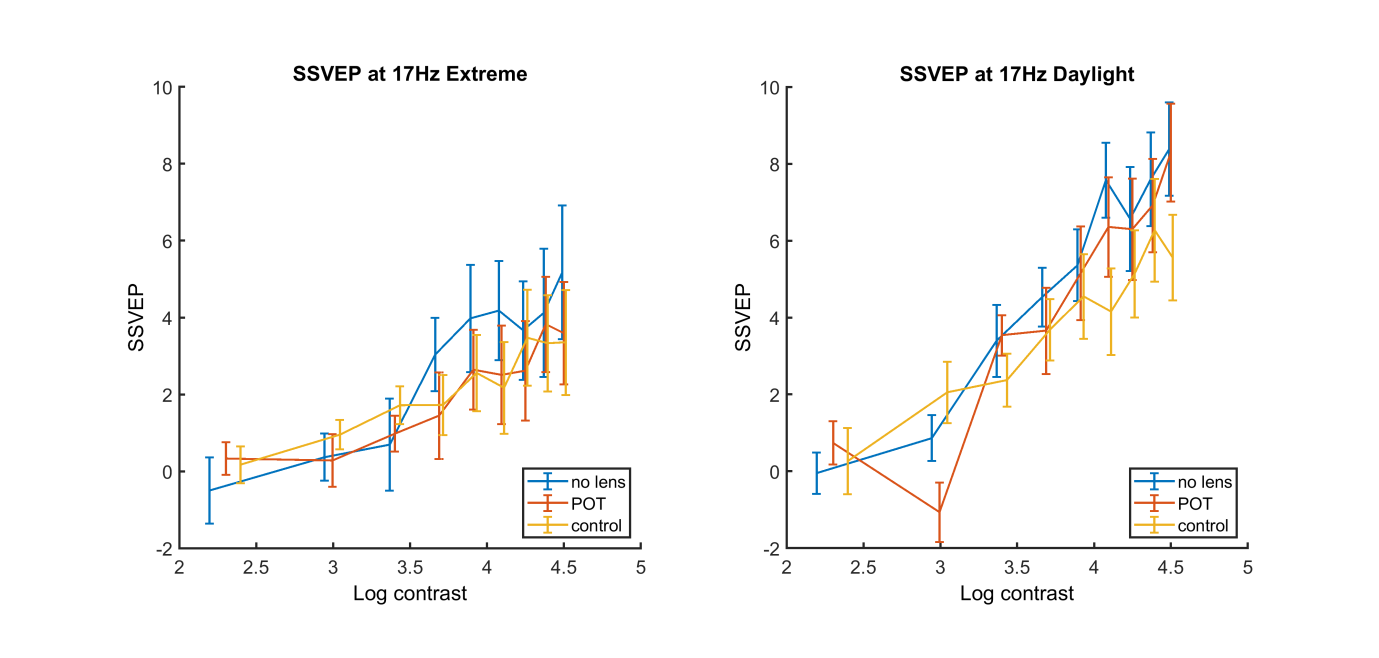


*Supplementary Figure 24: SSVEP against log contrast for no lens, POT and control lens conditions for (left) those who chose more saturated (extreme) colours and (right) those who chose colours closer to the daylight locus for the 17Hz stimulation in Experiment 2. Error bars indicate ± 1SE of the mean.*

As well as the SSVEP response, background EEG activity was analysed, a significant main effect model emerged, accounting for 90% of the variance. There was an effect of contrast - reduced background activity with increasing contrast. There was also a main effect of transmission. For a full table of results, see Supplementary Table 19.

*Supplementary Table 19: Results of the linear mixed effect model for background activity for 17Hz stimulation frequency. Those who chose extreme values (saturated colours) are compared to those who chose less saturated colours, closer to the daylight locus as a reference. The no lens condition is used as the reference for comparison for the POT and control lenses.*

|  | Coefficient estimate | Lower CI | Upper CI | p-value |
| --- | --- | --- | --- | --- |
| **Contrast** | **-146.40** | **-199.14** | **-93.66** | **<0.001** |
| Extreme | -27.97 | -393.44 | 337.50 | 0.881 |
| POT | -55.78 | -494.84 | 383.28 | 0.803 |
| Control lens | -147.98 | -702.21 | 406.24 | 0.600 |
| Transmission | -2.06 | -9.00 | 4.88 | 0.560 |

**6) Behavioural Results**

6.1) Assumptions of the Ordinal Model

Supplementary Figure 25 shows the histogram of the behavioural judgements. The distribution was skewed by 0.77. Therefore, an ordinal mixed effects model was created in R (R Core Team 2021) using the package “ordinal” (Christensen, 2023).


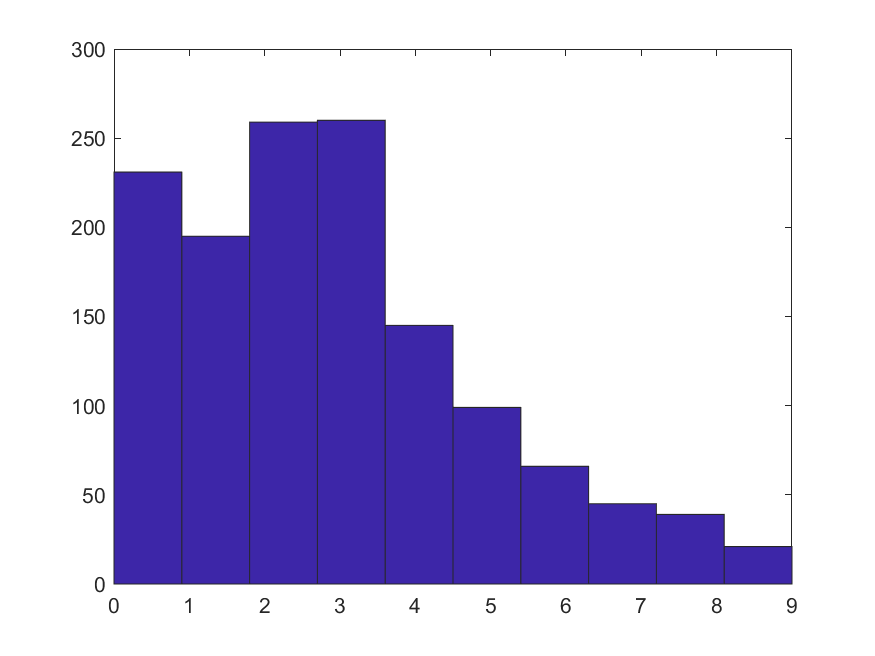


*Supplementary Figure 25: Histogram of the behavioural responses in Experiment 2 before transformation.*

6.2) Behavioural Results based on Distance from Daylight Locus

The same analysis as in the main manuscript was conducted to compare those who chose more saturated colours compared to those who chose colours closer to the daylight locus. This can be seen in Supplementary Figure 26.


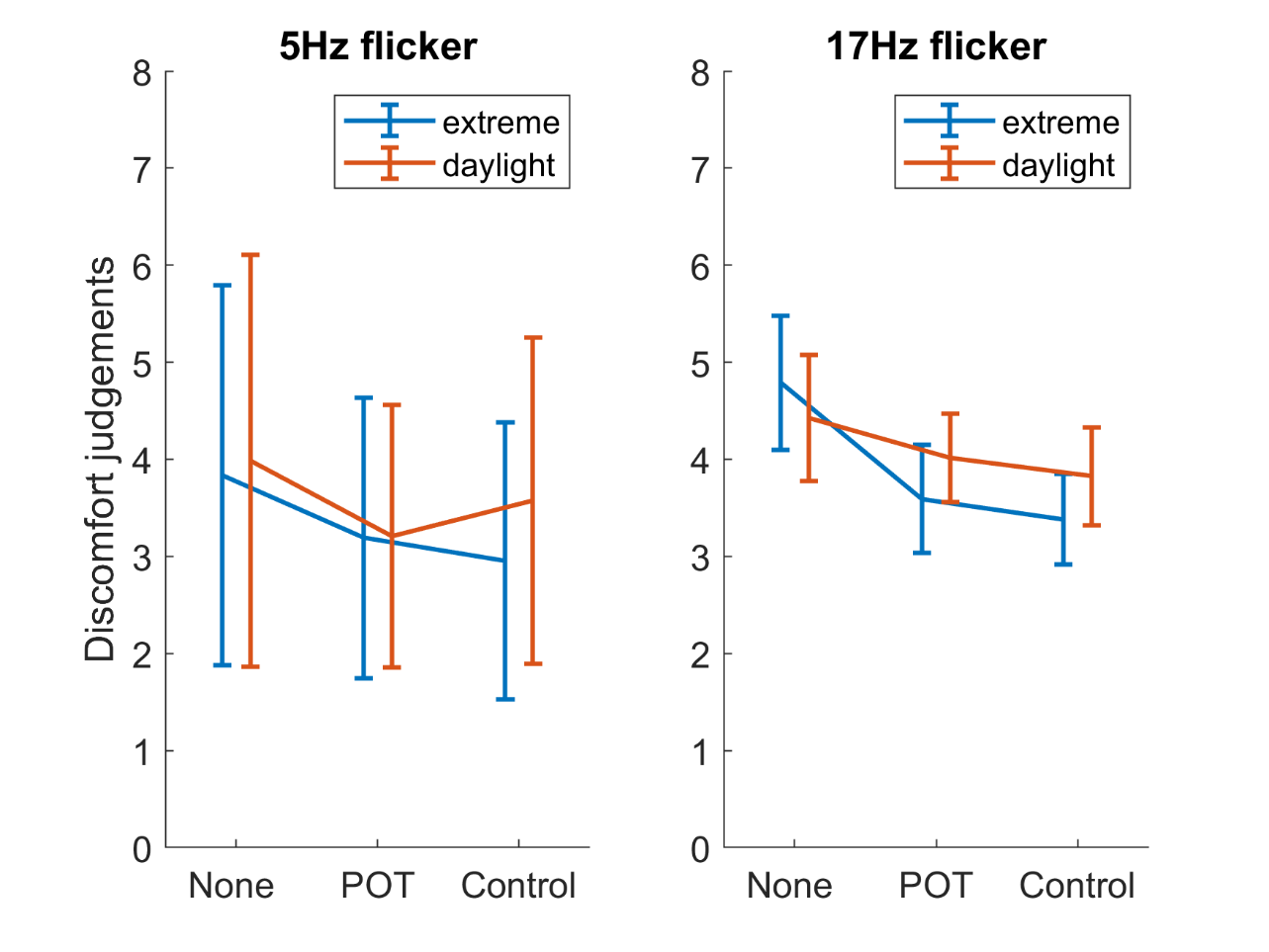


*Supplementary Figure 26: Behavioural results of the Experiment 2. Average discomfort judgements are plotted against lens condition for extreme and daylight groups. Please note data have been transformed by adding a scalar of +1 to all scores. Error bars are ±1 SE of the mean.*

Results of the ordinal mixed effect model can be seen in Supplementary Table 20. Results showed that faster flicker was judged to be more uncomfortable. There was also an effect of lens, for both POT and control lens there was reduced discomfort compared to no lens. There was also an effect of transmission, but no group difference, or any interaction.

*Supplementary Table 20: Results of the linear mixed effect model for behavioural responses. The group who chose more extreme saturated colours are compared to the group who chose the colours closer to the daylight locus as a reference. The no lens condition is used as the reference for comparison for the POT and control lenses. The 5Hz flicker rate is compared to the 17Hz flicker rate as a reference.*

|  | Coefficient estimate | p-value | Lower CI | Upper CI |
| --- | --- | --- | --- | --- |
| **POT** | **1.165** | **0.015** | **0.23** | **2.10** |
| **Control lens** | **1.93** | **0.003** | **0.65** | **3.21** |
| **Flicker** | **0.86** | **P < 0.001** | **0.39** | **1.33** |
| Extreme | 1.758 | 0.191 | -0.87 | 4.37 |
| **Transmission** | **0.03** | **P < 0.001** | **0.02** | **0.05** |
| POT x flicker | -0.05 | 0.881 | -0.71 | 0.61 |
| Control lens x flicker | -0.35 | 0.300 | -1.01 | 0.31 |
| Extreme x POT | 0.57 | 0.128 | -0.16 | 1.29 |
| Extreme x control lens | -0.60 | 0.085 | -1.29 | 0.08 |
| Extreme x flicker | 0.008 | 0.983 | -0.66 | 0.68 |
| Extreme x flicker x POT | -0.15 | 0.758 | -1.10 | 0.80 |
| Extreme x flicker x control lens | 0.12 | 0.802 | -0.84 | 1.09 |
